# Tumor Cell-Derived Exosomal circ-PRKCI Promotes Proliferation of Renal Cell Carcinoma via Regulating miR-545-3p/CCND1 Axis

**DOI:** 10.3390/cancers15010123

**Published:** 2022-12-25

**Authors:** Yiguan Qian, Yang Li, Luwei Xu, Ke Chen, Ning Liu, Xiaobing Yang, Qian Lv, Rongfei Li, Changcheng Zhou, Zheng Xu, Ruipeng Jia, Yu-Zheng Ge

**Affiliations:** 1 Department of Urology, Nanjing First Hospital, Nanjing Medical University, Nanjing 210012, China; 2 Department of Pathology, Nanjing First Hospital, Nanjing Medical University, Nanjing 210012, China

**Keywords:** renal cell carcinoma, exosome, circ-PRKCI, microRNA-545-3p, cyclin D1, proliferation

## Abstract

**Simple Summary:**

The morbidity and mortality of renal cell carcinoma (RCC) remain high, and there is an urgent need to explore the reliable biomarker and molecular mechanism of RCC. Circ-PRKCI is a novel circRNA discovered in recent years, and has been confirmed to participate in tumor progression by acting as an miRNA sponge. However, the role of circ-PRKCI in RCC remains unclear. Here, with the aid of an online bioinformatics database and luciferase reporter assays, we found that circ-PRKCI could bind to miR-545-3p, while CCND1 served as a target of miR-545-3p. Caki-1-derived exosomes could thus inhibit miR-545-3p expression via the upregulation of circ-PRKCI. Through a series of functional experiments, we confirmed that Caki-1-derived exosomal circ-PRKCI promoted RCC proliferation by regulating the miR-545-3p/CCND1 axis. Our study will provide new insights for RCC clinical treatment.

**Abstract:**

Renal cell carcinoma (RCC) originates from the epithelial cells of the renal tubules and has a high degree of malignancy and heterogeneity. Recent studies have found that exosomes regulate intercellular communication via transferring various bioactive molecules, such as circular RNAs (circRNAs), which are critical for cancer progression. However, the role of tumor cell-derived exosomal circRNAs in RCC remains unclear. In this study, we reported the high expression of circ-PRKCI in RCC tissues and serum exosomes. We also found that circ-PRKCI could be transferred exosomally from highly malignant RCC cells to relatively less malignant RCC cells. Tumor cell-derived exosomal circ-PRKCI promoted the proliferation, migration, and invasion of RCC cells, while inhibiting their apoptosis. Mechanistically, we found that circ-PRKCI promoted the proliferation of RCC via the miR-545-3p/CCND1 signaling pathway. Our study is the first to report the potential mechanisms of tumor cell-derived exosomal circ-PRKCI in RCC. In conclusion, this study will provide a new understanding about the molecular mechanisms of RCC progression.

## 1. Introduction

Renal cell carcinoma (RCC) originates from the epithelial cells of the renal tubules and has a high degree of malignancy [1]. In 2020, the incidence of RCC remained high, with new cases accounting for 5% and 3% of all cancers in men and women, respectively [2]. The lack of specific diagnostic biomarkers for RCC results in most patients having distant metastases at the time of initial diagnosis [3]. Although significant progress has been made in treatments such as surgical resection, immunotherapy, and targeted therapy in recent years, the overall prognosis of RCC patients remains unsatisfactory, particularly for those with metastatic RCC [4]. Therefore, there is an urgent need to explore potential markers and investigate the oncogenesis mechanisms of RCC.

Exosomes are lipid bilayer membrane vesicles about 30–150 nm in diameter that are secreted by most cells [5]. Exosomes are stable in various types of eukaryotic fluids and therefore assist in intercellular communication, which is essential for tumor development [6]. Moreover, studies have shown that highly aggressive tumor cells can deliver exosomes to target cells and promote tumor growth and metastasis [7]. For example, exosomes released by highly metastatic hepatocellular carcinoma (HCC) cells can enhance the metastasis and invasion of HCC cells with low metastasis potential [8]. A growing body of evidence has emerged to indicate that exosomes conduct intercellular communication via transferring different enriched molecules including proteins, lipids, and non-coding RNAs (ncRNAs) [9], while the role of exosomal ncRNAs has been challenged due to their relatively low level [10].

Circular RNAs (circRNAs) are a novel group of ncRNAs and form a closed-loop structure via trans-splicing without the 3′ and 5′ ends [11]. Because of their closed-loop structure, circRNAs are stable in tissues and body fluids, thus regulating gene expression at multiple levels of transcription, post-transcriptional modification, and translation [12]. In addition, circRNAs are enriched in exosomes and play an important role in intercellular communication. Exosomal circRNAs are stable in the blood and expected to be novel biomarkers for clinical detection [13]. For example, pancreatic cancer cells can promote endothelial permeability by delivering exosomal circ-IARS to human umbilical vein endothelial cells, thus accelerating the progression of pancreatic cancer [14].

Through the exoRBase database, we screened for circRNAs differentially expressed in plasma exosomes of various cancers and found that circ-PRKCI (circbase ID: hsa_circ_0067934) was upregulated in plasma exosomes of RCC patients. circ-PRKCI is a novel circRNA discovered in recent years which is located at chr3: 170013698–170015181 with a full length of 1483 bp. circ-PRKCI has been shown to participate in the progression of multiple tumors as competitive endogenous RNA (ceRNA) [15,16]. However, the role of circ-PRKCI in RCC remains unclear. This study provides the first report of the high circ-PRKCI expression in RCC tissues and serum exosomes. Additionally, the Caki-1 cell line, a metastatic cell derived from RCC, is the most malignant cell in RCC cell lines [17] and cloud release circ-PRKCI to RCC cells with low metastasis potential in an exosome manner thus enhanced the proliferation, migration, and invasion of RCC cells, while it inhibited their apoptosis. Mechanistically, we found that circ-PRKCI promoted the proliferation of RCC through the microRNA-545-3p (miR-545-3p)/cyclin D1 (CCND1) signaling pathway. Therefore, our study reveals the role of exosomal circ-PRKCI in the occurrence and development of RCC, providing a novel target for exosome-based RCC treatment.

## 2. Materials and Methods

### 2.1. Patient and Tissues Collection

After approval by the Ethics Committees of Nanjing First Hospital (NJFH; Nanjing Medical University, China), twenty-four pairs of cancer and peritumor normal tissue, and six blood samples from RCC patients underwent partial or radical nephrectomy at the department of urology in NJFH from January 2018 to December 2019 were collected. None of the patients received neoadjuvant chemotherapy or radiotherapy. Additionally, six blood samples from age- and gender-matched healthy volunteers were collected at a health examination center in NJFH. Informed consent was obtained from all participants, in agreement with the Declaration of Helsinki.

### 2.2. Cell Culture

Human RCC cell lines (786-O, ACHN, Caki-1, and 769-P) and the normal epithelial cell line HK-2 were obtained from Procell Life Science (Wuhan, China). RPMI-1640 medium (Thermo Scientific, Shanghai, China) was used to cultivate 786-O and 769-P cells. ACHN and HK-2 cells were cultured in Dulbecco’s Modified Eagle’s Medium (Thermo Scientific). McCoy’s 5A medium (Thermo Scientific) was used to cultivate Caki-1 cells. All medium is mixed with 10% fetal bovine plasma (FBS) (Thermo Scientific). All cell lines were kept in an environment with 5% CO_2_ at 37 °C.

### 2.3. Plasmid Construction and Transfection

To silence circ-PRKCI, short hairpin RNA (sh-RNA) against the circ-PRKCI gene was ligated into the GV493 vector (Genechem, Shanghai, China). To overexpress circ-PRKCI, full-length circ-PRKCI was ligated into a GV486 vector (Genechem). For the overexpression of CCND1, full-length CCND1 was ligated into a GV657 vector (Genechem). The miR-545-3p mimics and inhibitor were also obtained from Genechem. All plasmids have a corresponding negative control. After the cells were cultured at 50–70% confluence, Lipofectamine 3000 Transfection Reagent (Thermo Scientific) was used for transfection.

### 2.4. Isolation and Characterization of Exosomes

Two different methods to isolate exosomes were adopted in this study. The serum exosomes were isolated using ExoQuick Exosome Precipitation Solution (System Biosciences, Palo Alto, CA, USA). In brief, we added 63 μL ExoQuick Exosome Precipitation Solution to 250 μL human serum and incubated it at 4 °C overnight. After centrifugation at 1500× *g* for 30 min, the sediment became exosomes, and we used 1 × PBS to resuspend it. The cellular exosomes were isolated through differential ultracentrifugation [18]. Briefly, we dcentrifuge 50 mL of cell culture at 4 °C and 10,000× *g* for 20 min to obtain the cell culture supernatant. The supernatant was taken at 4 °C and centrifuged at 100,000× *g* for 60 min. We removed the supernatant and suspended the sediments in PBS, filtered them through a 0.22 μm cell strainer, and then centrifuged the liquid at 100,000× *g* for 60 min. The previous steps were repeated to obtain exosomes for subsequent experiments. In subsequent experiments, we resuspended 10 μg of exosomes in 100 μL of PBS and co-cultured them with 1 × 10^5^ cells in 6-well plate for 24 h.

To examine exosomes, the morphology of exosomes was determined via transmission electron microscopy. To estimate the exosomes size distribution and concentration, nanoparticle tracking analysis (NTA) was conducted using ZetaView (Particle Metrix, Meerbusch, Germany).

### 2.5. RNA Isolation, Reverse Transcription and Quantitative Real-Time Polymerase Chain Reaction (qRT-PCR)

We used TRIzol reagent (Tiangen, Beijing, China) to extract total RNA from RCC tissues, cells, or exosomes. The purity and concentration of the RNA was measured by a NanoDrop 2000 spectrophotometer (Allsheng, Hangzhou, China). To synthesize cDNA, we used RevertAid Reverse Transcriptase (Thermo Scientific) to conduct reverse transcription. A qRT-PCR was performed using a PerfectStart Green qPCR SuperMix (TransGen Biotech, Beijing, China) and the ABI Prism 7500 Detection System (Applied Biosystems, Waltham, MA, USA). The expression of circ-PRKCI, miR-545-3p, and CCND1 was calculated according to the 2^−ΔΔCt^ method. The primer sequences are listed in Table S1. GADPH and U6 were used as internal references.

### 2.6. Western Blotting

Total proteins in cells and exosomes were extracted using the RIPA protein lysate kit (Beyotime, Shanghai, China). After protein quantification by BCA kit (Beyotime), 10 μL of each group of proteins were taken for sodium dodecyl sulfate-polyacrylamide gel electrophoresis (SDS-PAGE). The proteins were then transferred to polyvinylidene fluoride (PVDF) membranes (Millipore, Billerica, MA, USA) after separation. After blocking, the PVDF membrane was co-incubated with the corresponding primary antibody overnight and then incubated with the secondary antibody for 1.5 h. Detailed information on these antibodies is provided in Table S2. An ECL system (Beyotime) was used for protein exposure. Original blots see Supplementary File S1.

### 2.7. Cell Counting Kit (CCK)-8 Assay

The cell viability of different groups was assessed by a CCK-8 kit (Dojindo, Kyushu, Japan). RCC cells (2000–3000 cells/well) were inoculated into 96-well plates after corresponding treatment and incubated for 0, 24, 48 and 72 h. Next, 10 μL CCK-8 and 100 μL complete medium were added to each well, and the cells were incubated at 37 °C with 5% CO_2_ for another 2 h. Finally, a microplate reader (Tecan, Männedorf, Switzerland) was used to measure the absorbance at 450 nm.

### 2.8. EdU Assay

Cell proliferation capacity was assessed by a Cell-Light^TM^ EdU Apollo^®^ 567 In Vitro Imaging Kit (Ribibio, Guangzhou, China). Transfected cells were plated onto 96-well plates at 1 × 10^4^ cells per well. After incubation at 37 °C for 24 h, the cells were incubated with 100 mM of EdU reagent at 37 °C for 2 h. After fixing the cells, we photographed them under a fluorescence microscope (Olympus, Tokyo, Japan).

### 2.9. Cell Apoptosis Assay

Treated cells were collected and resuspended in binding buffer. Cells were incubated for 30 min after adding 5 μL Annexin V-FITC (Beyotime), and then stained with 5 μL PI (Beyotime) for 5 min. The apoptosis rate was detected by flow cytometry (Beckman, Brea, CA, USA) and determined with the FlowJo software (version: 10.6.2).

### 2.10. Cell Scratch Assay

Treated cells were inoculated in 24-well plates at 2 × 10^5^ cells/well. The 1% serum-free medium was added when the cell growth fusion rate reached 60%. After the cell growth fusion rate reached 100%, the cells were scratched with a 10 μL sterile tip. The detached cells were washed off with PBS, and serum-free culture medium was added. The scratch width was observed and recorded under an inverted microscope (Nikon, Tokyo, Japan). After continuing the culture for 24 h, the scratch width was observed again under the microscope and recorded.

### 2.11. Transwell Assay

Cell invasion capacity was measured with Transwell chambers coated with Matrigel. Treated cells (2 × 10^4^) suspended in 200 μL of serum-free medium were added to the upper chamber, and 600 μL of complete medium was added to the well. All Transwell experiments were incubated in a 37 °C environment for 48 h. The infiltrated cells in the lower layer of the chambers were stained with crystal violet and photographed under an inverted microscope.

### 2.12. Animal Studies

For the subcutaneous tumorigenesis model, a total of 24 male immune-deficient BALB/c nude mice (4–5 weeks old) were purchased from Vital River Laboratories (Zhejiang, China) and were randomly divided into two groups (n = 3 for each group). First, 786-O cells were treated with exosomes or PBS. Then, we injected 5 × 10^6^ cells suspended in 200 μL of plasma-free medium in the dorsal flank region of each mouse. We monitored the tumor growth of each mice every week for 4 weeks. Finally, the mice were sacrificed, and the weight and volume of each subcutaneous tumor was measured.

For the metastasis model, six male immune-deficient BALB/c nude mice (4–5 weeks old) were randomly divided into two groups (n = 3 for each group). First, 786-O luciferase cells were treated with exosomes or PBS. We then injected 1 × 10^6^ cells suspended in 100 μL of plasma-free medium via tail vein injection. After 4 weeks, the nude mice were assessed for metastasis burden using the ECL system (Beyotime).

### 2.13. Hematoxylin-Eosin (H&E) Staining

The harvested tissues were fixed by 4% paraformaldehyde and embedded in paraffin. Tissues were then sliced into 5 μm sections and stained with HE, and were further analyzed by two experienced pathologists.

### 2.14. Subcellular Localization Assay

RCC cells were first washed with pre-cooled PBS and then treated with a cell separation buffer and a cell fragmentation buffer. We then used the Nuclear Extraction Kit (Beyotime) to isolate the cytoplasmic and nuclear components. circ-PRKCI content in the cytoplasmic and nuclear components was detected using qRT-PCR.

### 2.15. Dual-Luciferase Reporter Assay

Using pmirGLO as a vector, the binding sequences of circ-PRKCI and CCND1 to miR-545-3p and its mutant sequence were inserted into pmirGLO to construct dual-luciferase vectors (Gene Pharma, Shanghai, China), respectively. Luciferase constructs (0.1 µg) were co-transfected into 786-O and ACHN cells along with miR-545-3p mimics or miR-545-3p NC. We measured the luminescence intensity of firefly luciferase and Renilla luciferase separately using the dual-luciferase assay (Lux-T020; BioLight, Guangzhou, China), and the ratio of them can reflect the binding ability of circ-PRKCI and CCND1 to miR-545-3p.

### 2.16. RNA Immunoprecipitation (RIP) Assay

An RIP kit (Biosense, Guangzhou, China) was used to investigate the binding of RNAs and AGO2 proteins according to the manufacturer’s protocol. An IgG antibody served as a negative control. The immunoprecipitated RNAs were isolated with TRIzol reagent (Tiangen, Beijing, China) and analyzed using qRT-PCR.

### 2.17. Statistical Analysis

All experimental assays were performed in triplicate. Results were presented as the mean ± standard deviation (SD) and analyzed using GraphPad Prism (version 7.0; GraphPad Software, San Diego, CA, USA). Analysis between groups was performed using a Student’s *t*-test or a one-way analysis of variance, with a *p* value < 0.05 as the threshold of significance.

## 3. Results

### 3.1. circ-PRKCI Was Significantly Upregulated in RCC Tissues and Serum Exosomes

**Figure 1:**
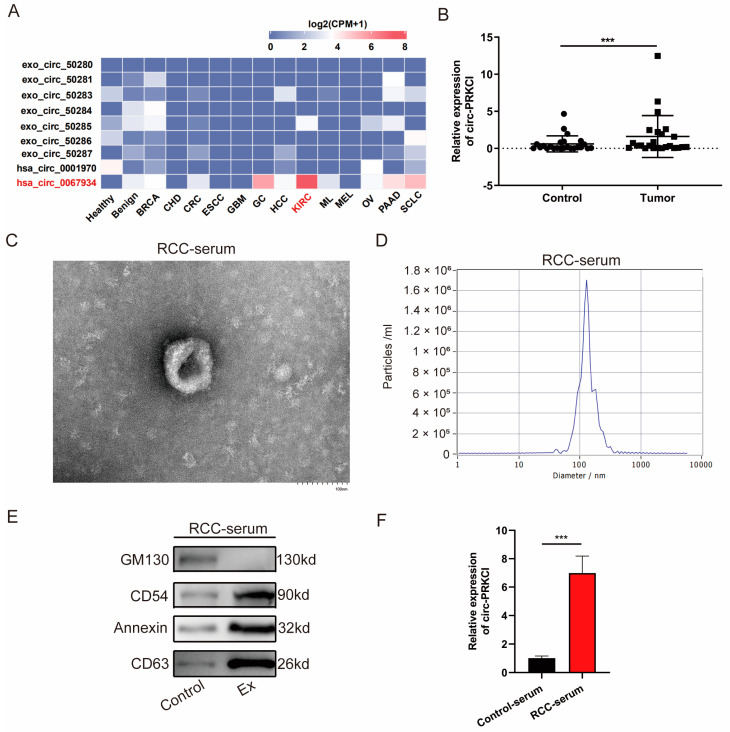
Exosomal circ-PRKCI was significantly upregulated in RCC tissues and serum. (**A**) The expression patterns of PRKCI related circRNAs in the plasma extracellular vesicles across cancer types in exoRBase database. (**B**) The relative expression of circ-PRKCI in RCC tissues and the adjacent non-malignant renal tissues. (**C**,**D**) Exosomes were isolated from RCC serum, and the morphology and size were examined by transmission electron microscopy (scale bar = 100 nm) and nanoparticle tracking analysis. (**E**) Protein expressions of GM130, CD54, Annexin, and CD63. (**F**) The relative expression of circ-PRKCI in exosomes isolated from RCC serum and healthy control serum. (*** *p* < 0.001).

For a comprehensive overview of PRKCI-related circRNAs in multiple cancer types, we explored their expression patterns in the exoRBase database and found that hsa_circ_0067934 (hereafter referred as circ-PRKCI) was upregulated in the plasma exosomes of RCC patients (Figure 1A). Although the difference did not reach the statistical significance level, circ-PRKCI has the potential to serve as a novel liquid biopsy biomarker with improved detection methods.

As shown in Figure 1B, the expression of circ-PRKCI was significantly upregulated in RCC tissues compared to that in matched peritumoral normal tissues. Furthermore, we isolated exosomes from RCC serum and characterized them by transmission electron microscopy, NTA, and Western blotting. The exosomes were round in shape, with a bilayer membrane and a diameter of 100~150 nm (Figure 1C,D). Western blotting indicated the positive expression of exosome-specific markers including CD54, annexin, and CD63 in exosomes, whereas they negatively expressed GM130, a negative exosomal marker (Figure 1E). Furthermore, qRT-PCR was used to quantify the circ-PRKCI expression level in the serum exosomes derived from RCC patients and healthy volunteers, and the expression levels of circ-PRKCI were dramatically increased (Figure 1F).

### 3.2. Intracellular Transferring of circ-PRKCI in an Exosome Manner

**Figure 2:**
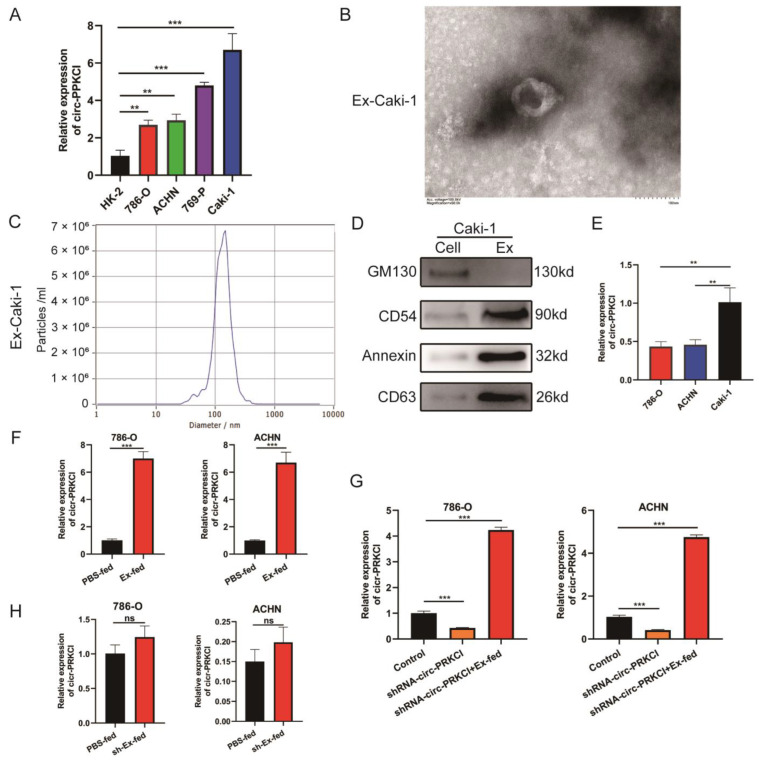
Intracellular transferring of circ-PRKCI in an exosome manner. (**A**) circ-PRKCI expression in RCC cell lines and HK-2 normal cells using qRT-PCR. (**B**,**C**) Exosomes were isolated from the supernatant of the culture medium of Caki-1 cells, and the morphology and size were detected by transmission electron microscopy (scale bar = 100 nm) and nanoparticle tracking analysis. (**D**) Protein expressions of exosomal markers GM130, CD54, Annexin, and CD63 in exosomes derived from the supernatant of the culture medium of Caki-1 cells and negative control Caki-1 cellular lysates. (**E**) circ-PRKCI expression in the exosomes derived from the supernatant of Caki-1, 786-O and ACHN cell cultures. (**F**) circ-PRKCI expression in 786-O and ACHN after being treated with exosomes derived from Caki-1. (**G**) circ-PRKCI expression in 786-O and ACHN after transfection with sh-NC or sh-circ-PRKCI either with or without the addition of Caki-1-derived. (**H**) circ-PRKCI expression in 786-O and ACHN after treatment with exosomes(sh-Ex) derived from the cell culture of sh-circ-PRKCI-Caki-1 cells. (** *p* < 0.01, *** *p* < 0.001, ns: no significance).

We first examined the circ-PRKCI expression level in RCC cell lines and the HK2 cell line. The qRT-PCR results showed that the expression of circ-PRKCI in RCC cell lines was higher than that in HK-2 cells, while the expression of circ-PRKCI was highest in the Caki-1 cell line and relatively lower in 786-O and ACHN cell lines (Figure 2A). The Caki-1 cell line is a metastatic cell derived from RCC and is the most malignant cell in RCC cell lines [17], while circ-PRKCI is upregulated in RCC tissues. We hypothesized that exosomes derived from Caki-1 cell culture could upregulate circ-PRKCI expression in 786-O and ACHN cells treated with exosomes. Thus, exosomes were isolated from the supernatant of Caki-1 cell cultures and examined by transmission electron microscopy and NTA. The results indicated that the exosomes were round, with a bilayer membrane and a diameter between 100~150 nm, as shown in Figure 2B,C. We also used western blotting to analyze the characteristics of the Caki-1 cell-derived exosomes. The results showed that the expressions of exosome-specific markers, including CD54, annexin, and CD63, were positive in exosomes, whereas they negatively expressed the exosome-negative marker GM130 (Figure 2D). Furthermore, we analyzed the circ-PRKCI expression level in the exosomes derived from the supernatant of Caki-1, 786-O and ACHN cell cultures, and found that circ-PRKCI was significantly enriched in exosomes derived form Caki-1 cells (Figure 2E).

Afterward, 786-O and ACHN cells were treated with exosomes derived from Caki-1, and the results of qRT-PCR indicated that exosomes derived from Caki-1 can upregulate the expression of circ-PRKCI (Figure 2F), which means that circ-PRKCI is obviously increased in 786-O and ACHN cells stimulated by exosomes, and that circ-PRKCI may be derived from exosomes derived from the Caki-1 cell culture. To further demonstrate this, we transfected shRNA-circ-PRKCI into 786-O and ACHN cells and the circ-PRKCI expression was significantly reduced (Figure 2G). However, after co-treatment with exosomes derived from the Caki-1 cell culture, the expression of circ-PRKCI was dramatically upregulated in RCC cells (Figure 2G). Furthermore, we transfected shRNA-circ-PRKCI plasmid into Caki-1 cells and isolated the exosomes (sh-Ex) from the cell culture of sh-circ-PRKCI-Caki-1 cells. We then treated 786-O and ACHN cells with sh-Ex and found that sh-Ex could not significantly regulate the expression of circ-PRKCI (Figure 2H). These results suggested that circ-PRKCI is mainly derived from exosomes, rather than from phenotypic changes within tumor cells.

### 3.3. Caki-1-Derived Exosomes Enhanced RCC Proliferation, Migration, Invasion, and Inhibited Apoptosis

**Figure 3:**
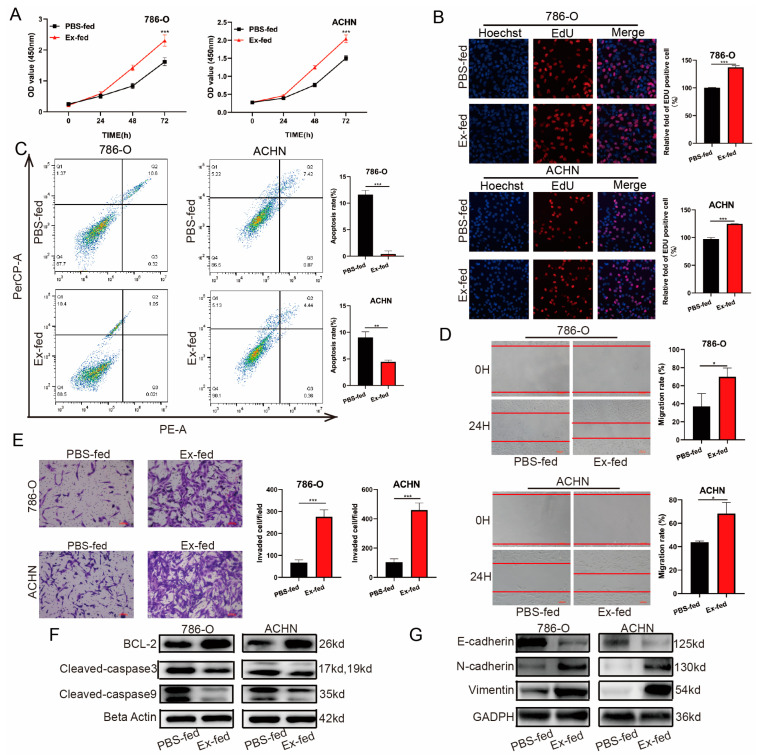
Caki-1-derived exosomes enhanced RCC proliferation, migration, invasion, and apoptosis inhibition. (**A**,**B**) Cell proliferation of 786-O and ACHN treated with PBS or Caki-1-derived exosomes was detected by a CCK8 and EdU assay. (**C**) Cell apoptosis was detected by flow cytometry analysis. (**D**) Cell scratch assay for migration ability of 786-O and ACHN transfected with PBS or Caki-1-derived exosomes. (**E**) Transwell assay for invasion ability of 786-O and ACHN transfected with PBS or Caki-1-derived exosomes. (**F**) Protein expression of representative apoptosis-related proteins in 786-O and ACHN treated with PBS or Caki-1-derived exosomes. (**G**) Protein expression of representative invasion-related proteins in 786-O and ACHN treated with PBS or Caki-1-derived exosomes. (* *p* < 0.05, ** *p* < 0.01, *** *p* < 0.001).

Growing evidence indicates that tumor cell-derived exosomes contribute to tumor growth and metastasis [19,20]. Thus, we explored the effect of exosomes derived from Caki-1 on the proliferation, apoptosis, migration, and invasion of 786-O and ACHN cells. The results of the CCK8 and EdU assays indicated that exosomes markedly promoted cell growth in exosome-stimulated RCC cells (Figure 3A,B). Next, we detected cell apoptosis using flow cytometry. As shown in Figure 3C, apoptosis was significantly inhibited in these two cells relative to those in the control groups. Additionally, cell scratch and Transwell assays revealed that exosomes derived from Caki-1 cell culture could significantly enhance the migration and invasion of the RCC cells compared to the PBS-fed group (Figure 3D,E). Additionally, the results of the Western blot indicated that the protein expression of BCL-2, N-cadherin, and vimentin was upregulated, whereas Cleaved-caspase 3, Cleaved-caspase 9, and E-cadherin expression was downregulated in RCC cells treated with exosomes (Figure 3F,G).

To explore whether circ-PRKCI could contribute to the phenotype changes of RCC cells treated with exosomes, we upregulated the expression of circ-PRKCI in 786-O and ACHN cells. CCK8 and EdU assay results indicated that cell proliferation ability was remarkably enhanced by circ-PRKCI (Figure S1A,B). Results of flow cytometry showed significant apoptosis inhibition by circ-PRKCI (Figure S1C). In addition, migration and invasion of RCC cells were enhanced by circ-PRKCI according to cell scratch and Transwell assays (Figure S1D,E).

To further detect at which level these effects of exosomes is attributable to circ-PRKCI, we treated 786-O and ACHN cells with sh-Ex. CCK8 and EdU assay results showed that the cell proliferation ability of the sh-Ex-fed group was slightly higher than the PBS-fed group, but there was no statistical difference (Figure S2A,B). Similarly, sh-Ex had little effect on cell apoptosis (Figure S2C). Furthermore, the results of the cell scratch and Transwell assays also indicated that the migration and invasion of the sh-Ex-fed group was slightly higher than the PBS-fed group, while the difference did not reach statistical significance (Figure S2D,E).

### 3.4. Caki-1-Derived Exosomes Could Promote Tumorigenicity and Metastasis of RCC In Vivo

**Figure 4:**
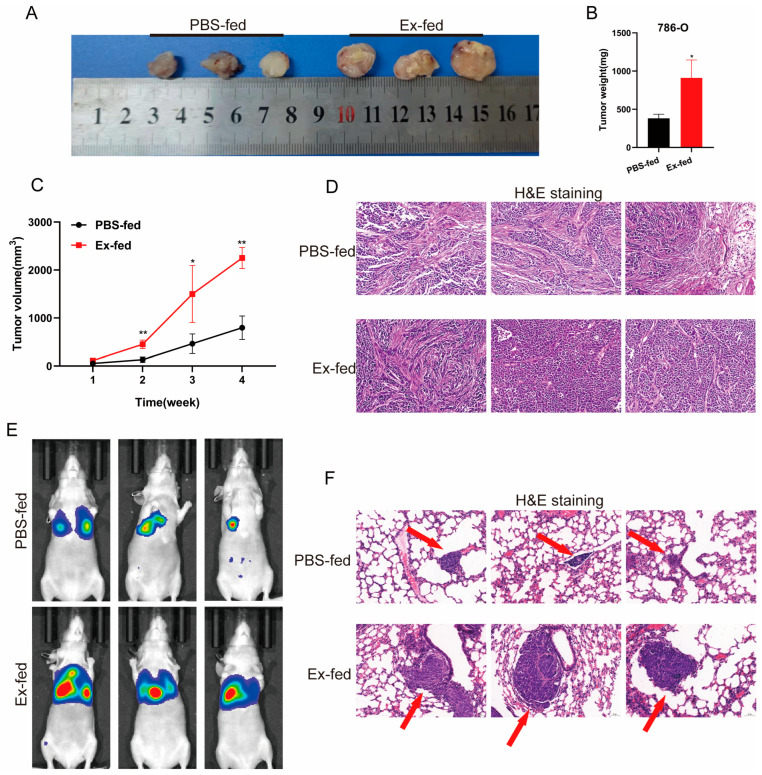
Caki-1-derived exosomes could promote the tumorigenicity and metastasis of RCC in vivo. (**A**) Tumors collected from nude mice were exhibited (n = 3 per group). (**B**) The tumor weight of PBS or exosomes treatment groups were measured and analyzed. (**C**) The tumor volume curve of the PBS or exosomes treatment groups were measured and analyzed. (**D**) Representative H&E staining of xenograft tumors in the PBS-fed or Ex-fed group. Scale bar, 50 μm. (**E**) Representative images of nude mice injected with PBS or exosomes treated 786-O cells via the tail vein (n = 3 per group). (**F**) Representative H&E staining of lung tissues in metastasis models. Scale bar, 50 μm. (* *p* < 0.05, ** *p* < 0.01).

In vivo experiments were performed to further verify the role of exosomes in RCC progression. First, we used the generation of xenografts to explore whether exosomes regulated the tumorigenicity of RCC in vivo. The results showed that the tumors derived from cells treated with exosomes were larger than those in the control groups (Figure 4A), in terms of both tumor weight and volume (Figure 4B,C). The H&E staining of xenograft tumors is shown in Figure 4D. We then used a metastasis model to explore the effects of the exosomes. As shown in Figure 4E, obvious bioluminescence signals in the lungs injected with 786-O treated with exosomes indicated that exosomes derived from Caki-1 cells significantly enhanced the metastasis of 786-O cells. In line with this, H&E staining showed considerably more and larger nodules in the lungs of mice injected with 786-O treated with exosomes than in the PBS-fed group (Figure 4F). Overall, these results suggested that Caki-1-derived exosomes promote the tumorigenicity and metastasis of RCC in vivo.

### 3.5. circ-PRKCI Could Serve as a Sponge for miR-545-3p

**Figure 5:**
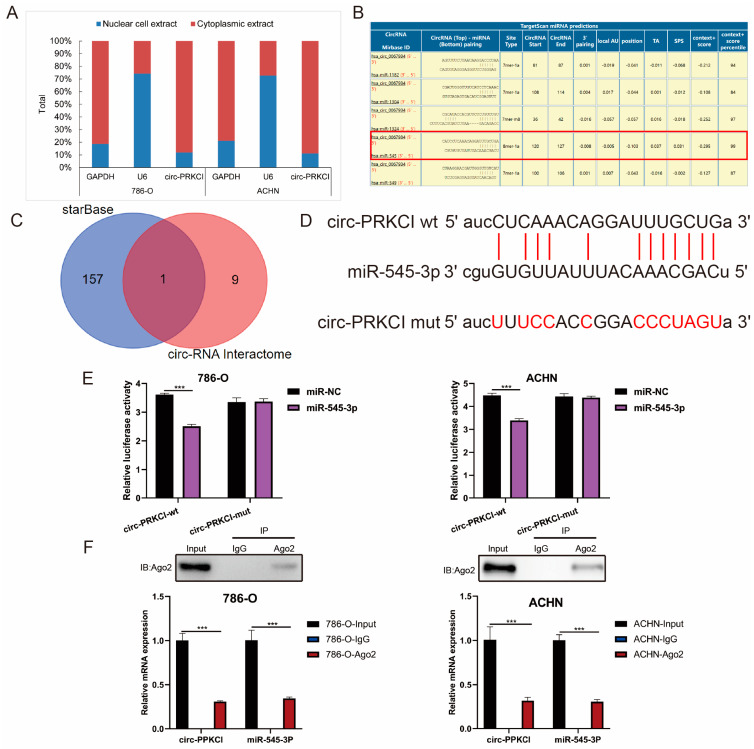
circ-PRKCI could serve as a sponge for miR-545-3p. (**A**) The subcellular distribution of circ-PRKCI in RCC cells, as detected by subcellular fraction assay. (**B**) The potential miRNAs linked to circ-PRKCI (circBase ID: hsa_circ_0067934) via online bioinformatics database Circular RNA Interactome, the selected hsa-miR-545 was highlighted within red frame. (**C**) A Venn diagram of the potential miRNAs linked to circ-PRKCI between Circular RNA Interactome and StarBase 3.0 database. (**D**) Schematic representation of the 3′-UTR of circ-PRKCI with the predicted target site for miR-545-3p. (**E**) The binging ability between miR-545-3p and circ-PRKCI was certified by Luciferase reporter assay. (**F**) An RIP assay was used to confirm the combination between miR-545-3p and circ-PRKCI. Top, Western blot results of IP efficiency of Ago2-antibody. Bottom, relative RNA expression compared with input. IgG served as a negative control. (*** *p* < 0.001).

It has been widely demonstrated that circRNAs could act as a sponge for miRNAs to exert their biological role when they are mainly concentrated in the cytoplasm [21]. The subcellular fraction assay demonstrated that the circ-PRKCI was mainly distributed in the cytoplasm of 786-O and ACHN cells (Figure 5A). Subsequently, we used the online bioinformatics database Circular RNA Interactome and StarBase 3.0 to predict the potential miRNAs linked to circ-PRKCI. Among these miRNAs, miR-545-3p displayed the best binding capacity and was the only intersection of the two databases (Figure 5B,C). We also found a binding site between miR-545-3p and circ-PRKCI, as shown in Figure 5D. A Luciferase reporter assay was performed to verify the combined relationship between miR-545-3p and circ-PRKCI. The results showed that miR-545-3p mimics inhibited the Luciferase activity of the wild-type reporter for circ-PRKCI compared with the group transfected with miR-NC (Figure 5E). We also found that circ-PRKCI and miR-545-3p could both be enriched by beads coated with anti-Ago2 compared with anti-IgG (Figure 5F). These findings indicated that miR-545-3p may be the target of circ-PRKCI.

### 3.6. CCND1 Was a Target of miR-545-3p

**Figure 6:**
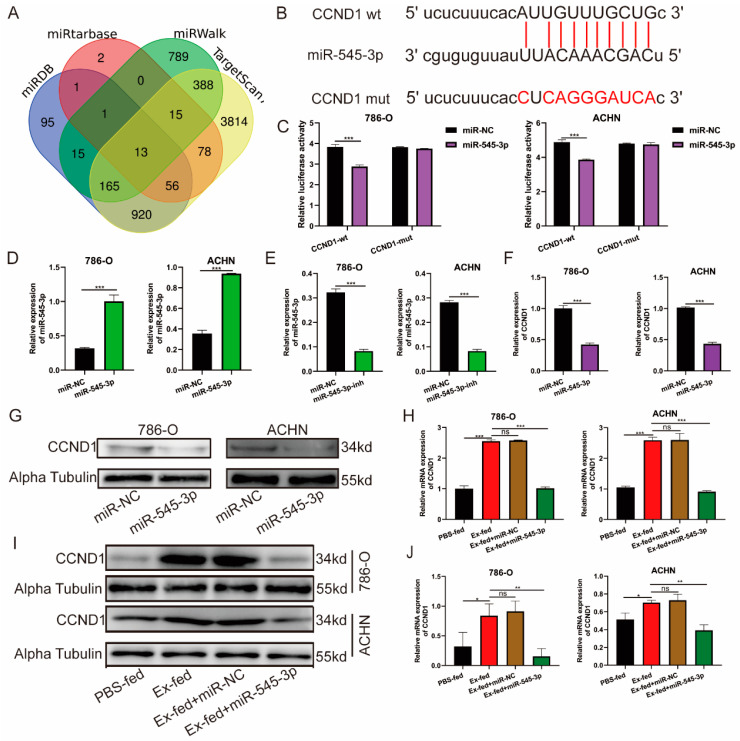
CCND1 was a target of miR-545-3p. (**A**) A Venn diagram of the target genes binding to miR-545-3p between the miRDB, miRtarbase, miRWalk, and TargetScan databases. (**B**) Schematic representation of the 3′-UTR of CCND1 with the predicted target site for miR-545-3p. (**C**) The binging ability between miR-545-3p and CCND1 was certified by Luciferase reporter assay. (**D**) The relative expression of miR-545-3p in 786-O and ACHN after transfection with miR-545-3p NC or mimics. (**E**) The relative expression of miR-545-3p in 786-O and ACHN after transfection with miR-545-3p NC or inhibitor. (**F**) The relative expression of CCND1 in 786-O and ACHN after transfection with miR-545-3p NC or mimics. (**G**) The protein level of CCND1 in 786-O and ACHN after transfection with miR-545-3p NC or mimics. (**H**) CCND1 expression in 786-O and ACHN after treatment with Caki-1-derived exosomes, along with miR-545-3p NC or mimics. (**I**) CCND1 protein level in 786-O and ACHN after treatment with Caki-1-derived exosomes, along with miR-545-3p NC or mimics. (**J**) The gray value statistical analysis results. (* *p* < 0.05, ** *p* < 0.01, *** *p* < 0.001, ns: no significance).

Many studies have demonstrated that miRNAs can silence target genes by binding to the 3′-UTR of target genes, which is essential for biological processes [22]. As shown in Figure 6A, four online bioinformatics databases (miRDB, miRtarbase, miRWalk, and TargetScan) were used to predict the target genes binding to miR-545-3p. We selected the CCND1 gene from the intersection of the four databases (Table S3), which has a binding site with miR-545-3p (Figure 6B). We used a luciferase reporter with either wild-type or mutant CCND1 3′-UTR sequences to further certify the interaction between miR-545-3p and CCND1. The results showed that miR-545-3p-mimics effectively reduced relative luciferase activity in 786-O and ACHN cells transfected with wild-type constructs, although there was no significant change in the mutant group (Figure 6C). We transfected miR-545-3p mimics, inhibitor, and corresponding negative controls into 786-O and ACHN cells. The qRT-PCR proved that mimics could upregulate the expression of miR-545-3p, while the inhibitor decreased it (Figure 6D,E). Furthermore, the results of qRT-PCR and Western blotting indicated that transfection with miR-545-3p mimics significantly decreased the mRNA and protein levels of CCND1 compared to that in the miR-NC groups (Figure 6F,G). To further confirm the regulatory effect of miR-545-3p on CCND1, we added miR-545-3p mimics to transfect RCC cells treated with exosomes. qRT-PCR and western blot results showed that the mRNA and protein levels of CCND1 were significantly enhanced in RCC cells treated with exosomes, while the effects were efficiently inhibited after co-treatment with miR-545-3p mimics (Figure 6H–J). Thus, CCND1 is a target of miR-545-3p.

### 3.7. circ-PRCKI Promoted RCC Proliferation by Regulating miR-545-3p/CCND1 Axis

**Figure 7:**
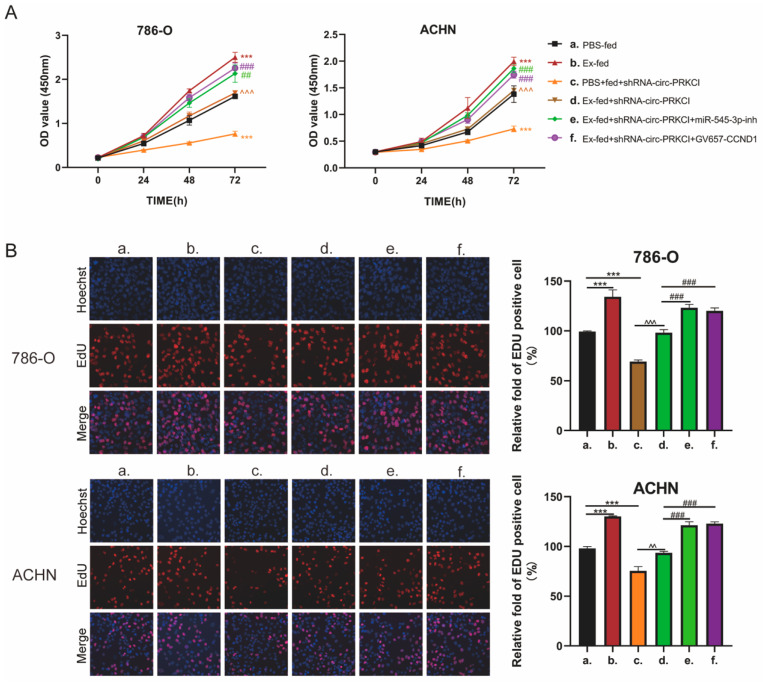
circ-PRCKI promoted cell proliferation by regulating the miR-545-3p/CCND1 axis. (**A**) Cell proliferation of 786-O and ACHN cells treated with (a) PBS; (b) Caki-1-derived exosomes; (c) PBS plus circ-PRKCI shRNA (shRNA-circ-ORKCI); (d) Caki-1-derived exosomes plus shRNA-circ-PRKCI; (e) Caki-1-derived exosomes and shRNA-circ-PRKCI plus miR-545-3p inhibitor (miR-545-3p-inh); (f) Caki-1-derived exosomes and shRNA-circ-PRKCI plus GV657-CCND1, as detected by CCK8 assay. (**B**) EdU assay for proliferation ability of 786-O and ACHN after the indicated treatment. (*** *p* < 0.001, compared to PBS-fed group; ^^^^ *p* < 0.05, ^^^^^ *p* < 0.001, compared to shRNA-circ-PRKCI group; ^##^ *p* < 0.05, ^###^ *p* < 0.001, compared to Ex-fed+shRNA-circ-PRKCI group).

We then investigated whether circ-PRKCI positively regulates malignant phenotypes of RCC cells via the miR-545-3p/CCND1 signaling pathway, and a series of rescue experiments in 786-O and ACHN cells were conducted. CCK8 results indicated that Caki-1-derived exosomes remarkably enhanced cell proliferation compared to those in the control group, while knockdown of circ-PRKCI inhibited cell proliferation. In the Ex-fed group, co-treatment with shRNA-circ-PRKCI effectively decreased the ability of cell growth, while this effect was diminished after co-treatment with miR-545-3p-inhibitor or GV657-CCND1 (Figure 7B). In addition, the EdU assay achieved similar results (Figure 7B). However, the downregulation of miR-545-3p or the upregulation of CCND1 had no effect on RCC cell migration, invasion, or apoptosis. We hypothesized that Caki-1 derived exosomes may influence RCC cell migration, invasion, and apoptosis through other pathways instead of the miR-545-3p/CCND1 axis. In summary, the above results proved that circ-PRCKI promoted RCC proliferation by regulating the miR-545-3p/CCND1 axis.

## 4. Discussion

CircRNAs have been widely studied, and exosomal circRNAs have been described as valuable biomarkers and therapeutic targets in different cancer types [23,24]. In this study, we screened a novel circRNA (circ-PRKCI) in the plasma exosomes of RCC patients from the exoRBase database. We were the first to report the high expression of circ-PRKCI in RCC tissues and serum exosomes, which can be transferred via exosomes. Furthermore, Caki-1-derived exosomes could promote RCC proliferation, migration, and invasion, and inhibited cell apoptosis. Mechanistically, circ-PRKCI promoted RCC proliferation via the miR-545-3p/CCND1 signaling pathway.

Circ-PRKCI is a novel ncRNA discovered in recent years and has been confirmed to participate in tumor progression by acting as miRNA sponges. For example, circ-PRKCI could sponge miR-3680-3p to stimulate the migration and proliferation of esophageal squamous cell carcinoma cells [16]. Accumulated evidence indicates that exosomes contain a specific cargo of ncRNAs to exert intercellular communication functions, thus affecting the occurrence and development of tumors [9]. However, controversies exist in the field, as the effect of exosomal ncRNAs in cell-to-cell communication has been doubted due to the low level, which urgently needs further investigation and verification [10]. Recently, different exosomal circRNAs have been reported to participate in tumor progression, especially circRNAs [23,24]. However, the role of circ-PRKCI in RCC remains unclear, and the role and mechanism of exosomal circ-PRKCI in tumors has not been reported.

miR-545-3p have been widely studied because of their roles in tumor progression. For instance, miR-545-3p enrichment could suppress colorectal cancer cell malignant behaviors by depleting MYO6 expression [25]. The downregulation of miR-545-3p promotes tumorigenesis and decreases radiosensitivity in neuroblastoma cells by upregulating the expression of hexokinase 2 [26]. CCND1 is a main regulator of the cell cycle [27], and has been reported to be involved in the cell proliferation of various cancers [28,29,30]. For example, the overexpression of CCND1 promoted cell proliferation of laryngeal squamous cell carcinoma [29]. In addition, CCND1 could interact with other factors to act as a proliferation promoter of colon cancer [30]. However, to date, there have been no studies on the miR-545-3p/CCND1 signaling pathways in RCC. With the aid of an online bioinformatics database and Luciferase reporter assays, we found that circ-PRKCI could bind to miR-545-3p, while CCND1 served as a target of miR-545-3p. Caki-1-derived exosomes could thus inhibit miR-545-3p expression via the upregulation of circ-PRKCI. Through a series of functional experiments, we confirmed that Caki-1-derived exosomal circ-PRKCI promoted RCC proliferation by regulating the miR-545-3p/CCND1 axis. However, themiR-545-3p/CCND1 pathway did not significantly affect the migration, invasion, and apoptosis of RCC cells.

Some limitations of the current study should be addressed. First, this study initially did not perform high throughput circRNAs screening, which lacked a comprehensive and unbiased view of circRNAs expression profiles in RCC. Second, circRNAs have been reported to regulate tumor progression via encoding peptides or interacting with selected protients [31,32]. In this study, we explored the ceRNA mechanism due to the subcellular localization of circ-PRKCI and subsequent bioinformatics analysis, which might hinder the full understanding of the underlying mechanisms of circ-PRKCI in RCC. Third, the diagnostic performance of serum exosomal circ-PRKCI has been explored in our center with limited sample size, which requires further validation in multiple centers with larger sample sizes with various cancer types. Fourth, more animal experiments with refined protocols such as adding sh-Ex as a control and direct injection with exosomes in animal experiments are warranted, which could make the conclusion more convincing. Finally, we did not explore the mechanism of Caki-1 derived exosomes affecting RCC migration, invasion, and apoptosis, which requires further exploration.

## 5. Conclusions

In summary, this is the first report on the oncogenic role of circ-PRKCI in RCC via the miR-545-3p/CCND1 axis, with a hope to offer a new idea for exosome-based RCC treatment.

## Data Availability

All the original data of this study are available from the corresponding author upon request.

## Supplementary Materials

The following supporting information can be downloaded at: https://www.mdpi.com/article/10.3390/cancers15010123/s1. Supplementary Table S1. The primer sequences of genes. Supplementary Table S2. Information of antibodies. Supplementary Table S3. Thirteen intersective genes between four databases. Supplementary Figure S1. Overexpression of circ-PRKCI enhanced RCC proliferation, migration, invasion, and apoptosis inhibition. Supplementary Figure S2. Little effect of sh-Ex on RCC proliferation, apoptosis, migration and invasion. File S1: Original blots.
