# Supplementary material for: Tumor Cell-Derived Exosomal circ-PRKCI Promotes Proliferation of Renal Cell Carcinoma via Regulating miR-545-3p/CCND1 Axis
Source: Cancers (Basel). 2022 Dec 25;15(1):123. doi: 10.3390/cancers15010123 (PMC9817713; doi:10.3390/cancers15010123)

Figure 1E

Annexin

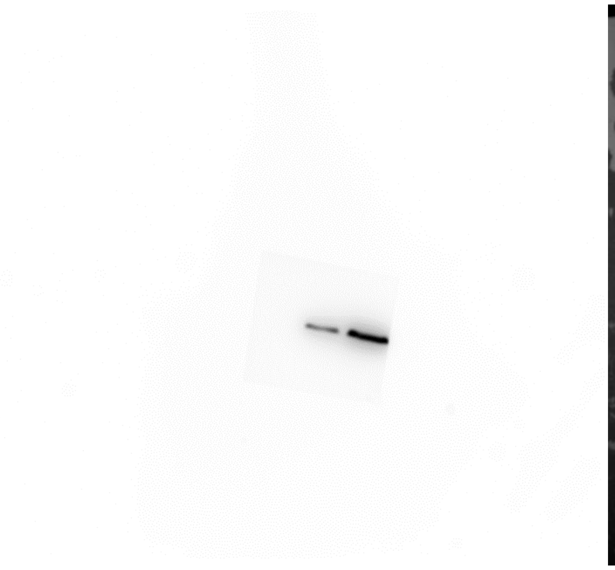

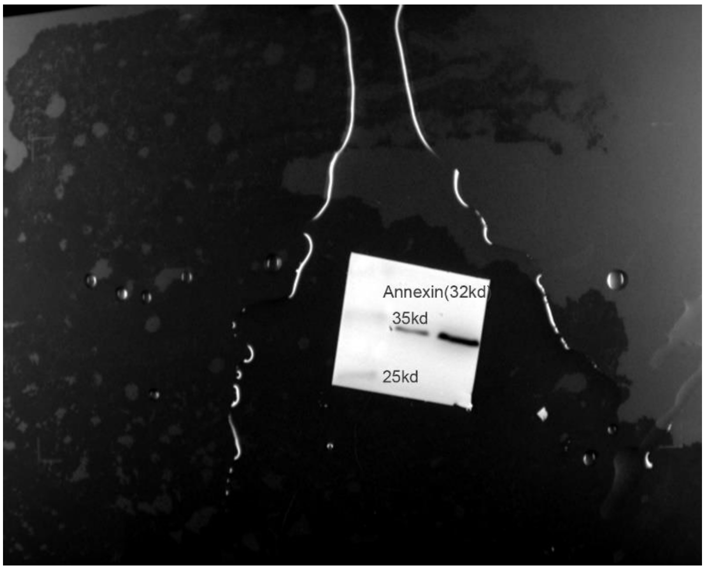

CD54

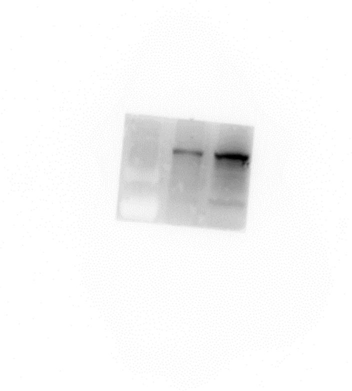

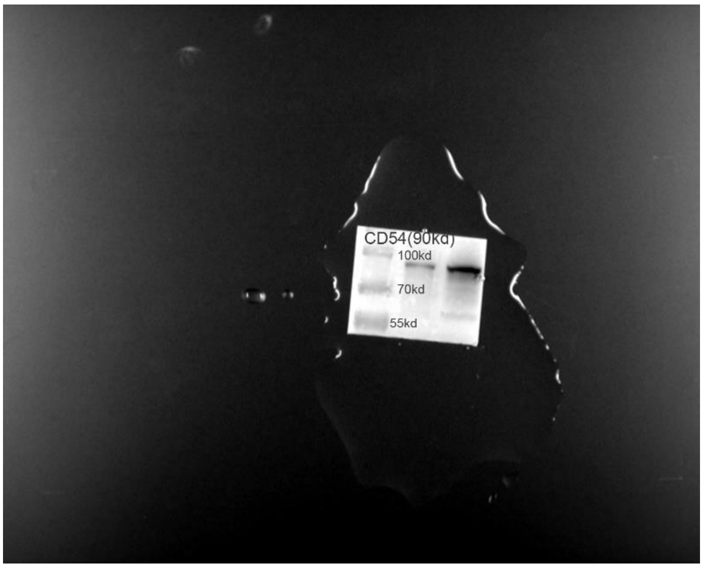

CD63

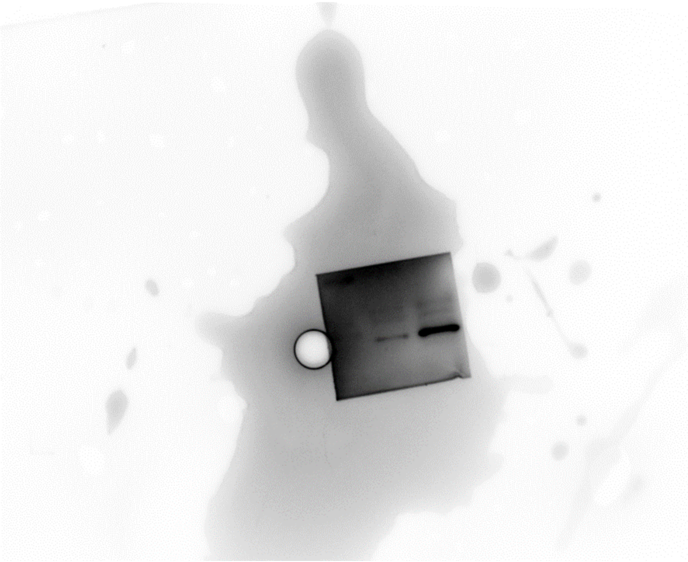

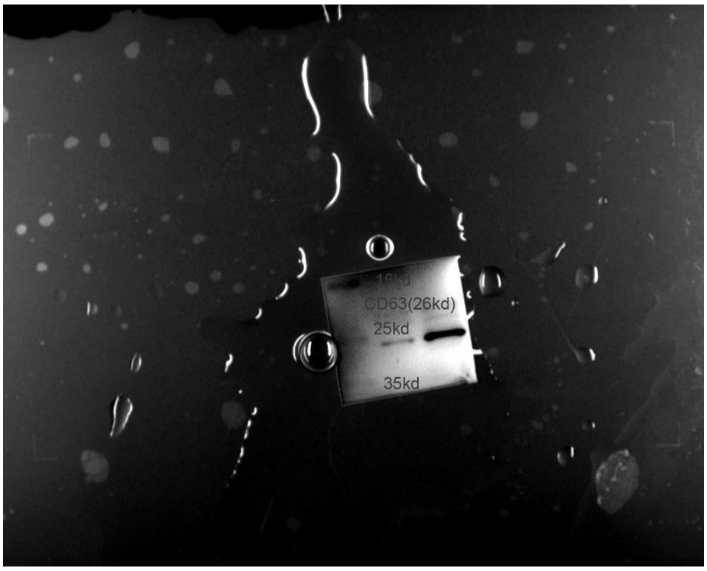

GM130

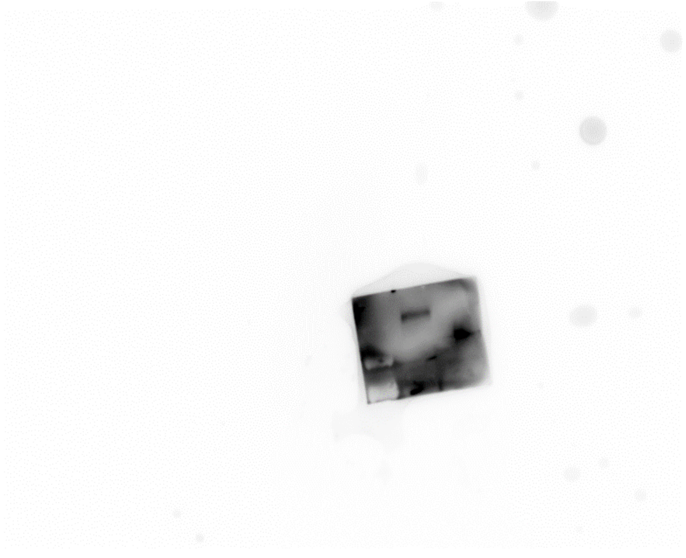

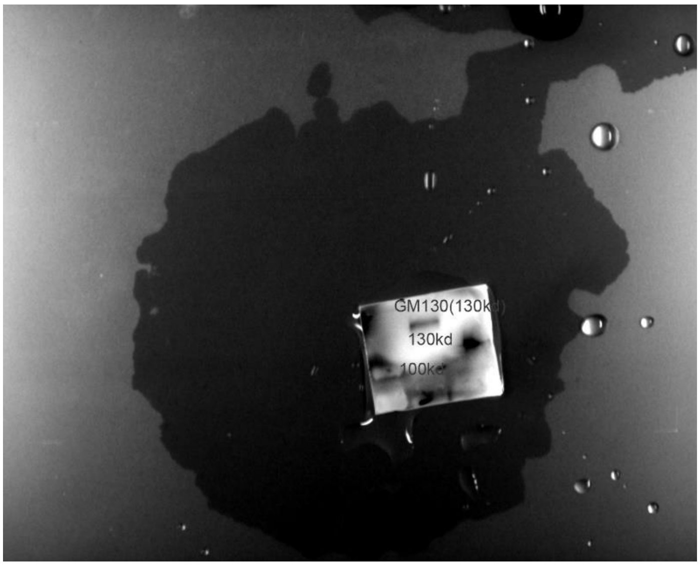

Figure 2D

Annexin

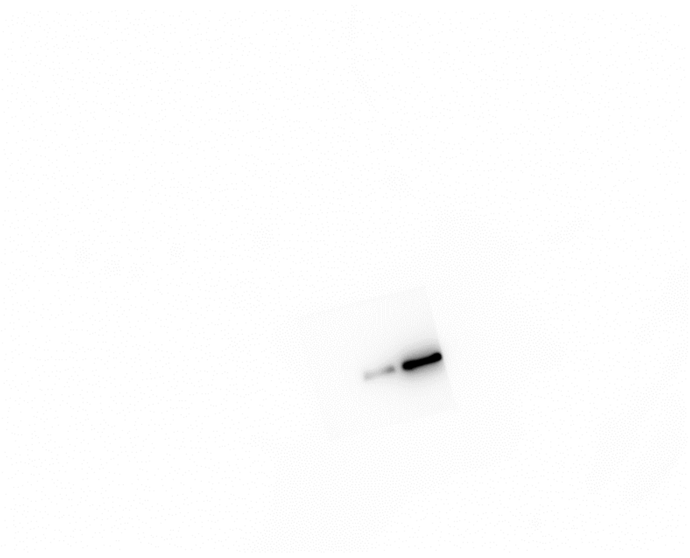

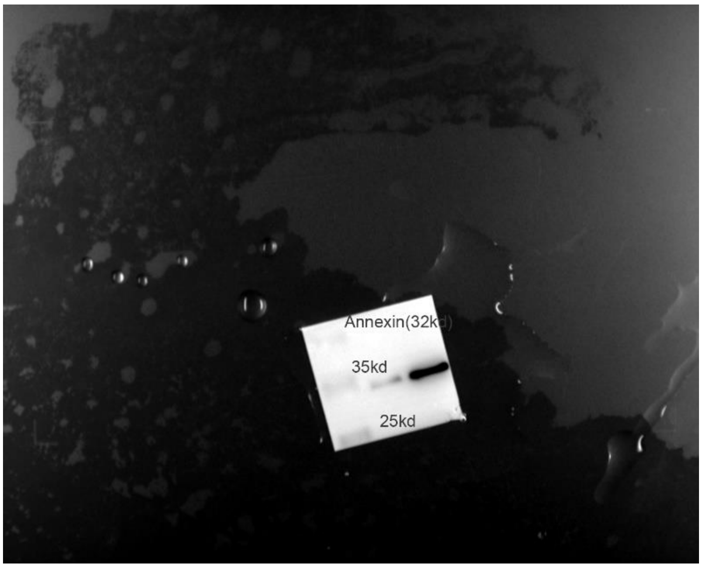

CD54

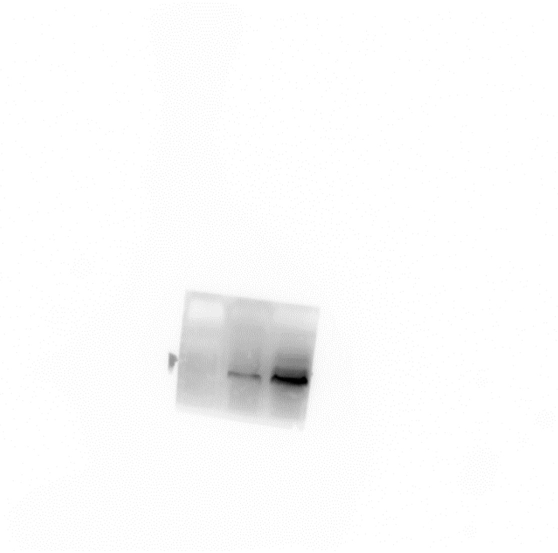

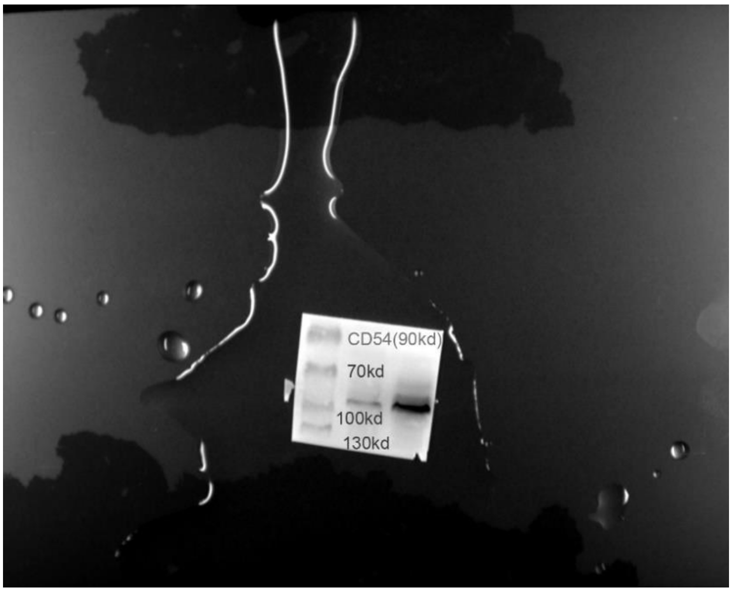

CD63

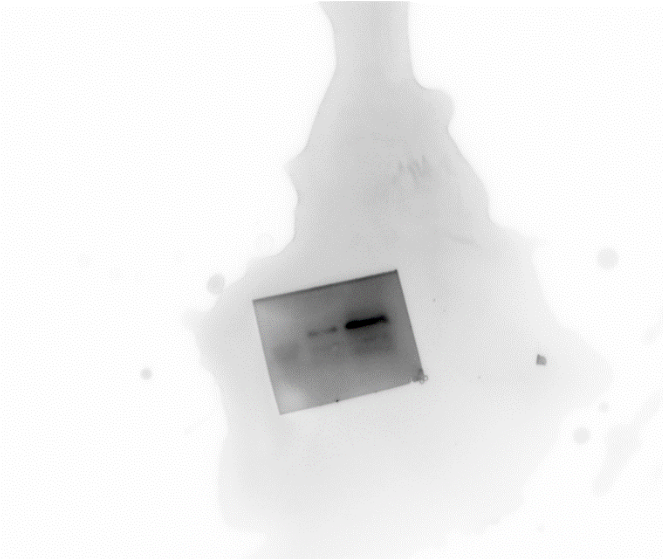

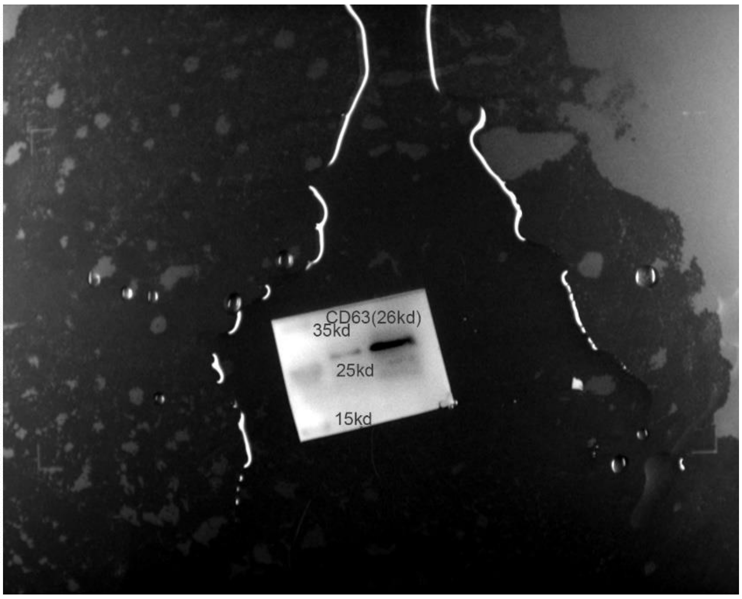

GM130

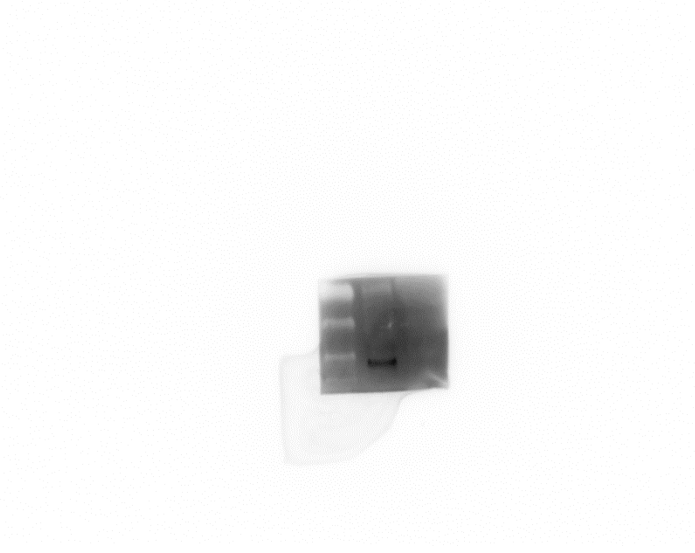

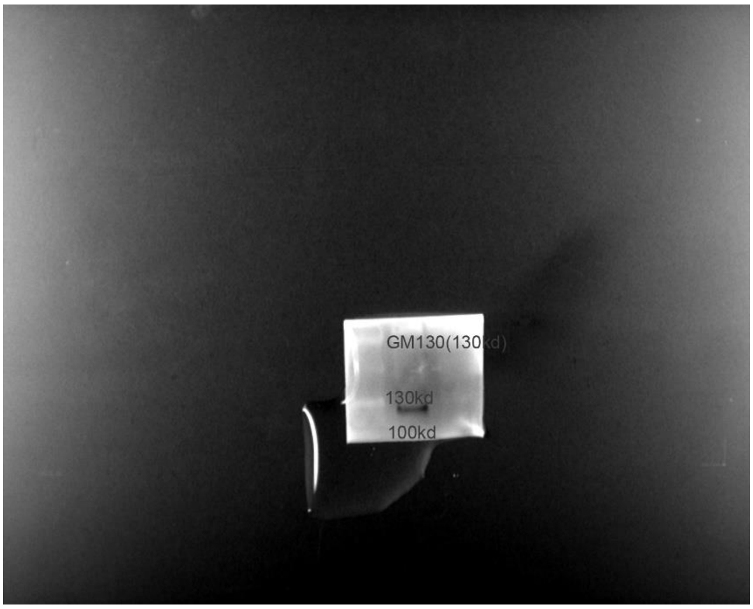

Figure 3F

786-O Beta Actin

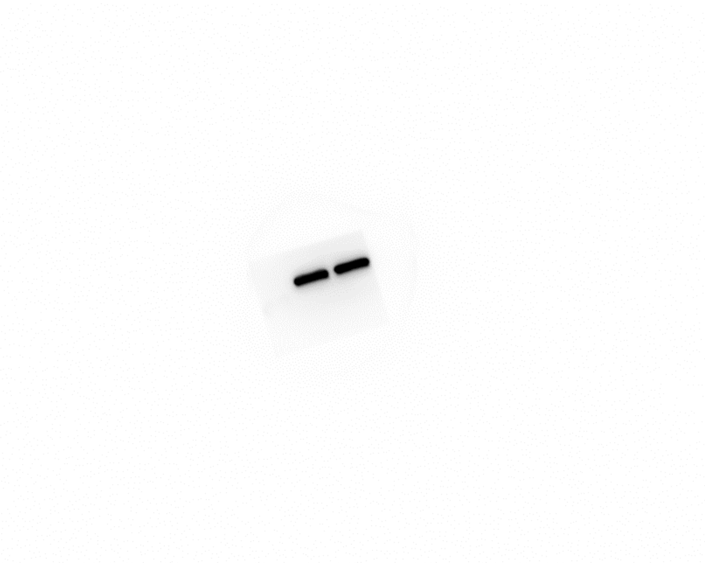

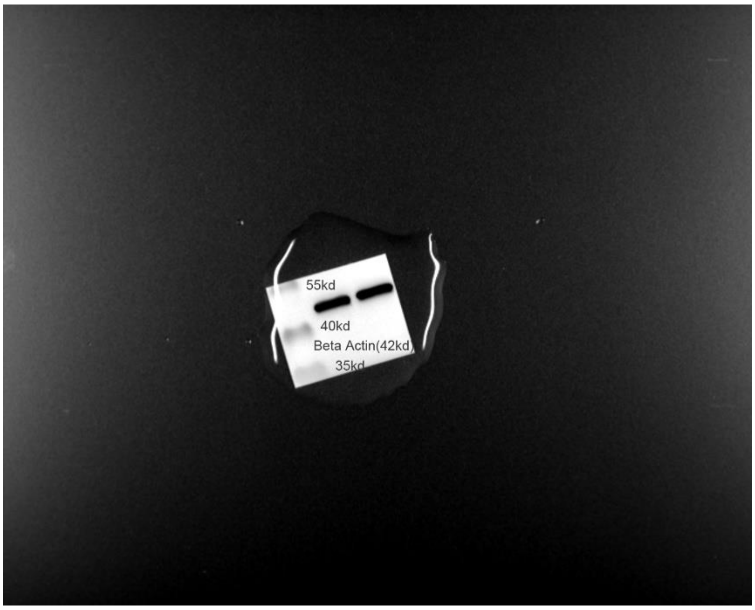

786-O BCL-2

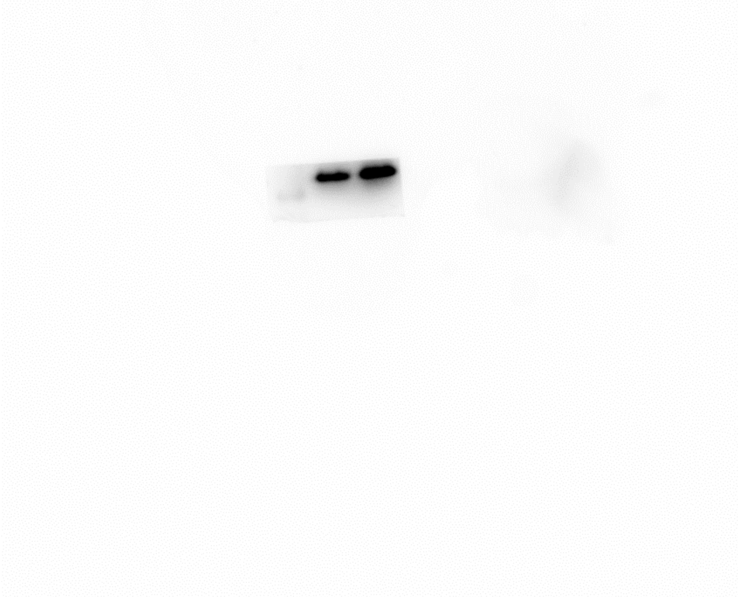

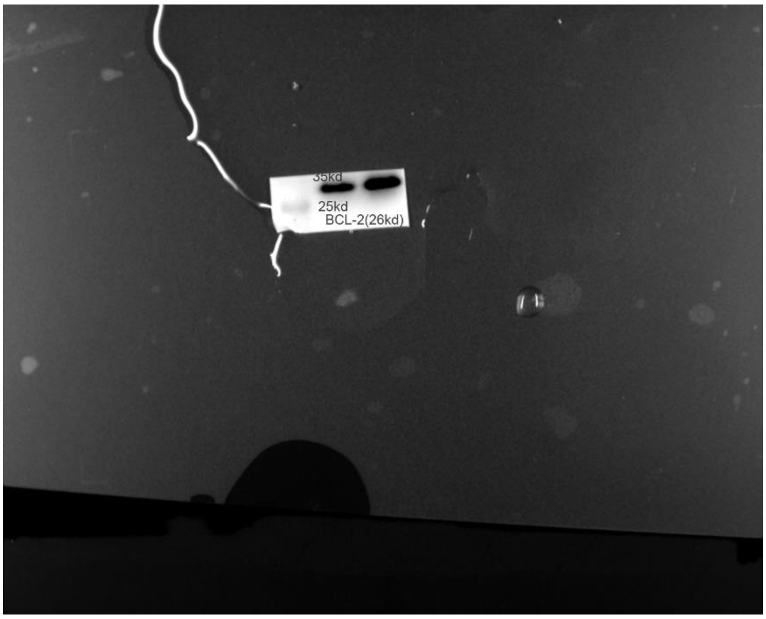

786-O Cleaved-caspase3

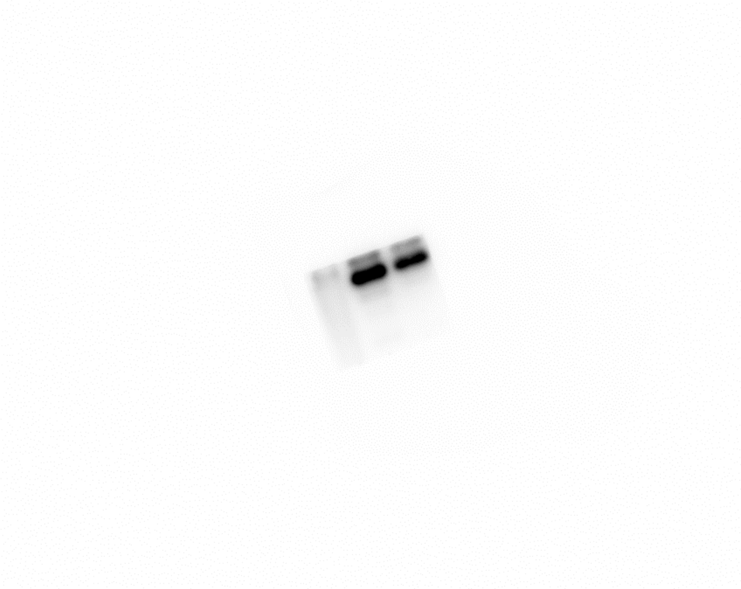

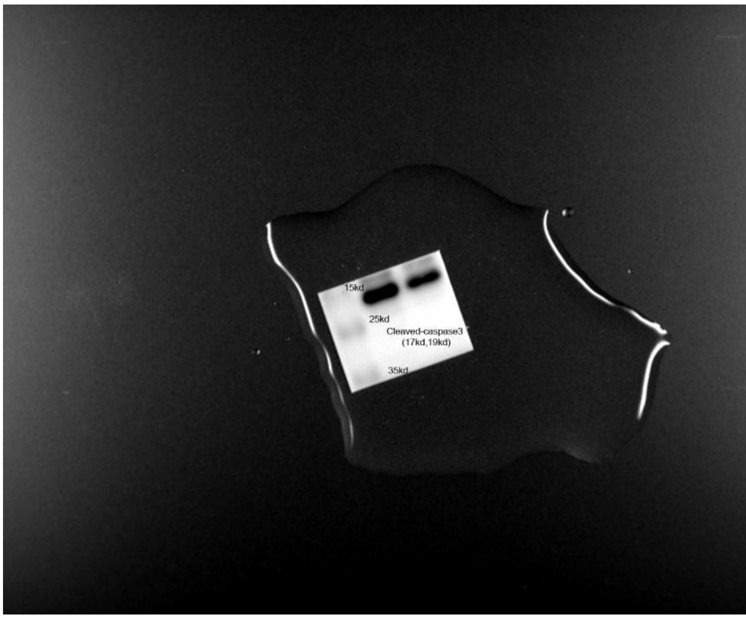

786-O Cleaved-caspase9

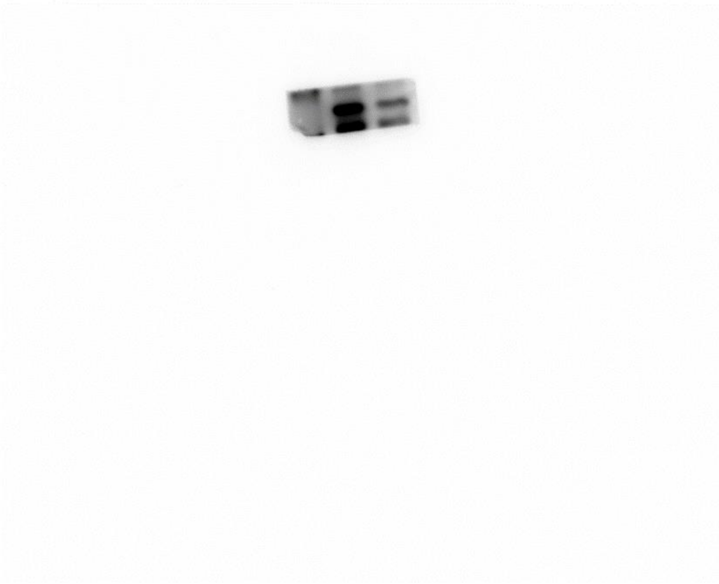

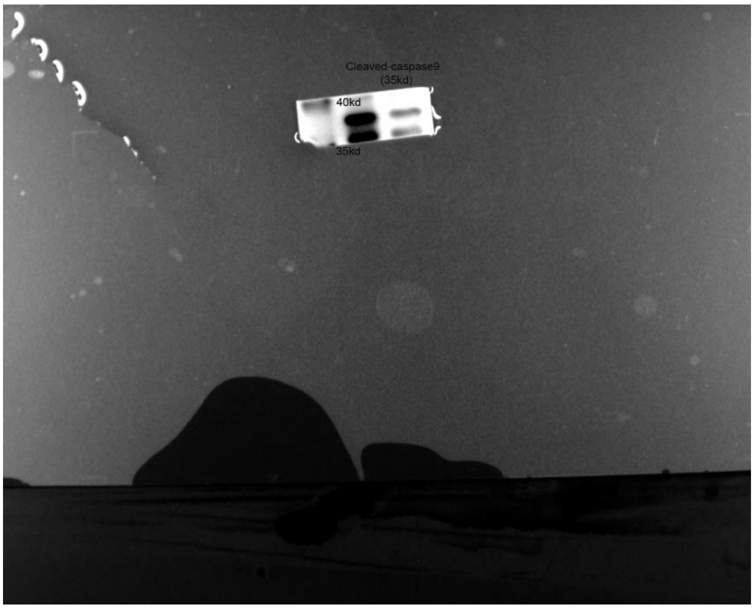

ACHN Beta Actin

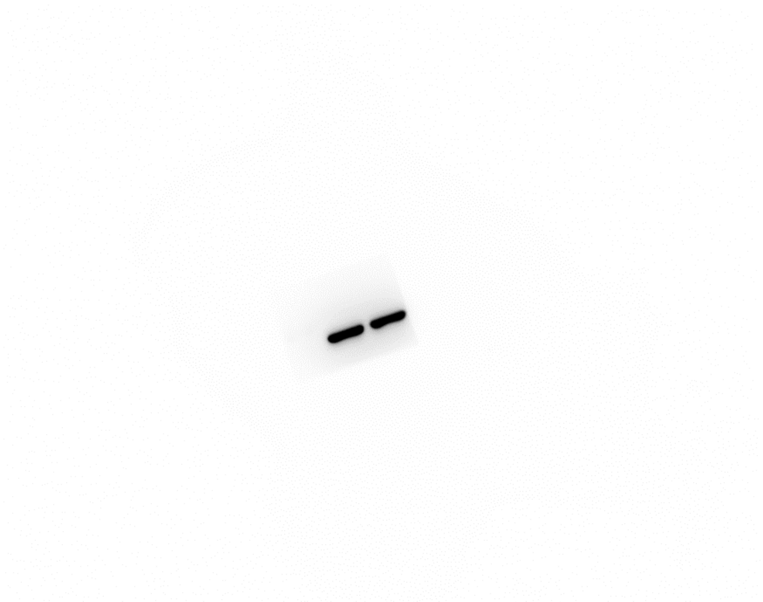

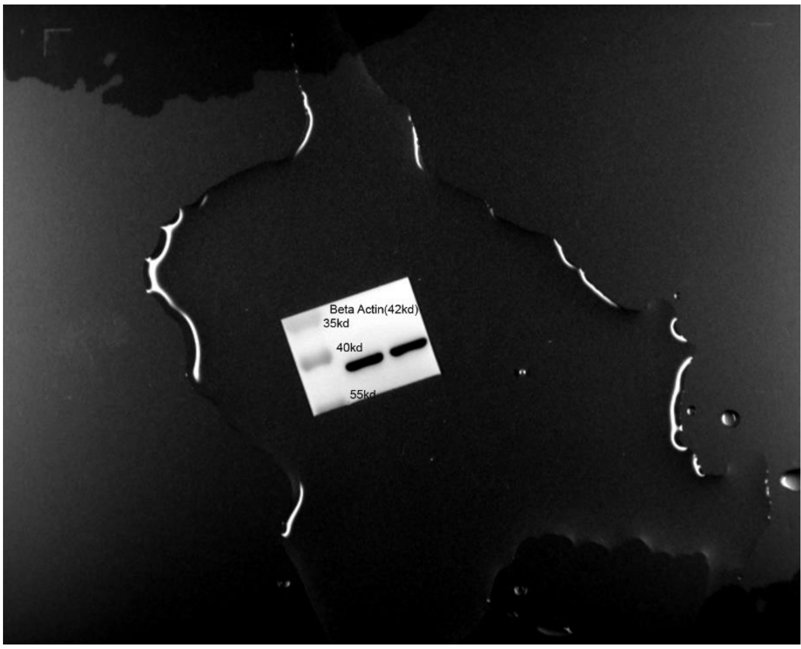

ACHN BCL-2

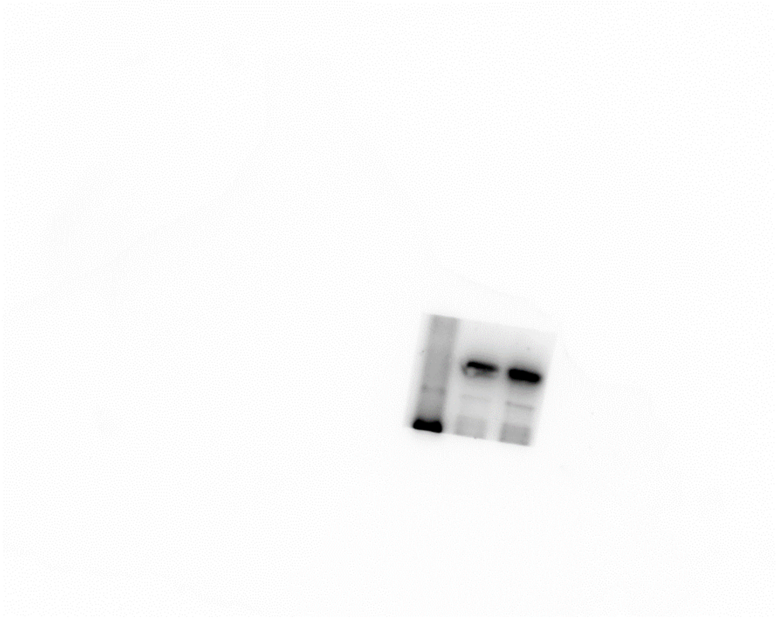

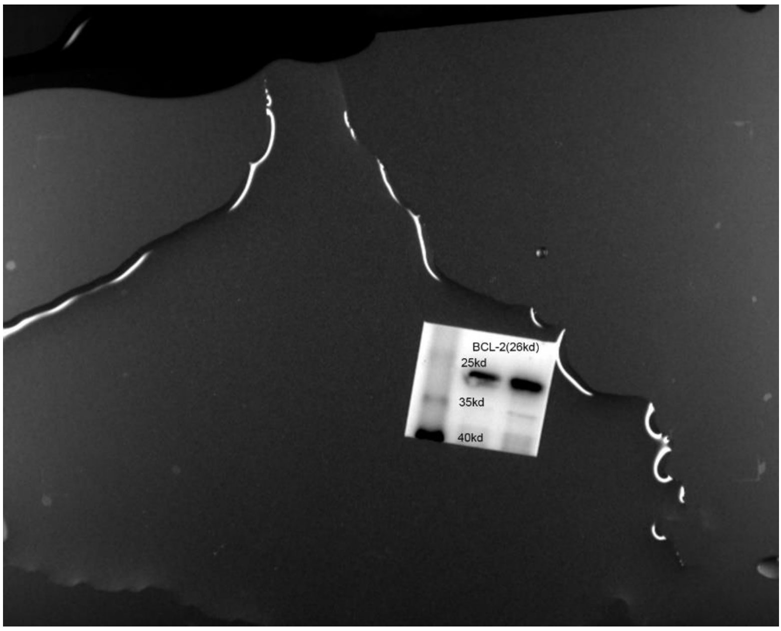

ACHN Cleaved-caspase3

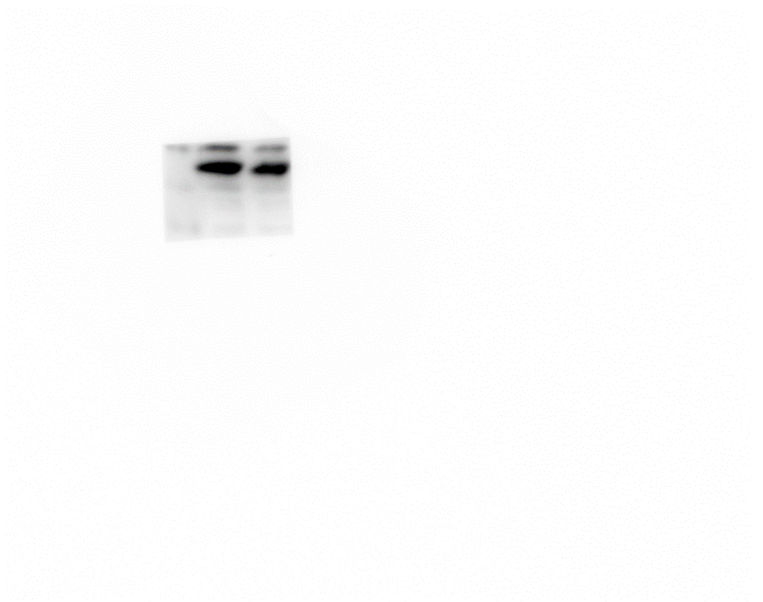

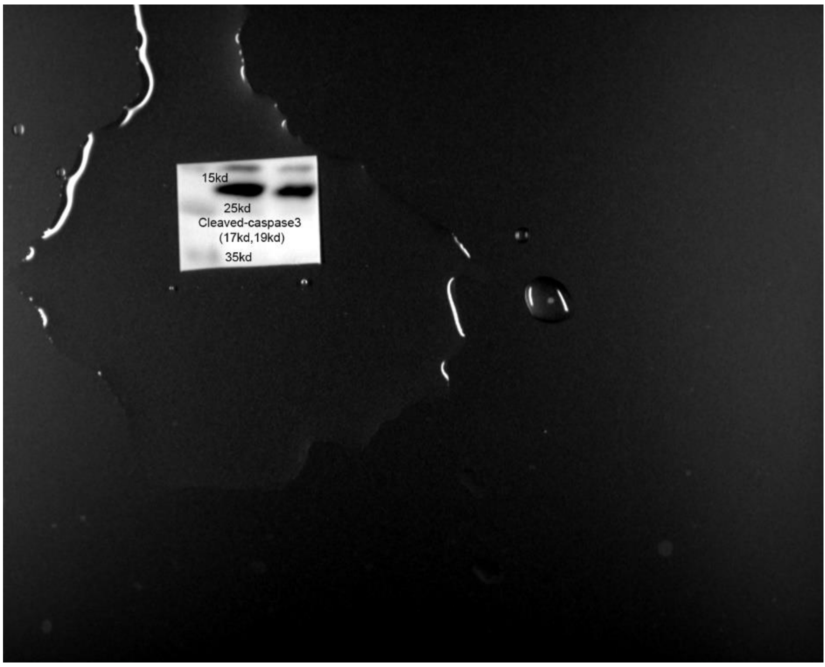

ACHN Cleaved-caspase9

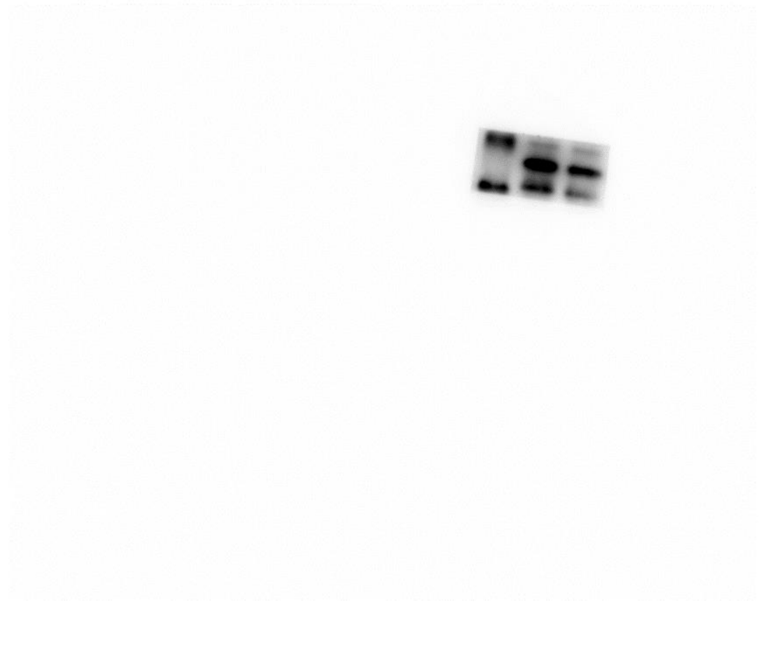

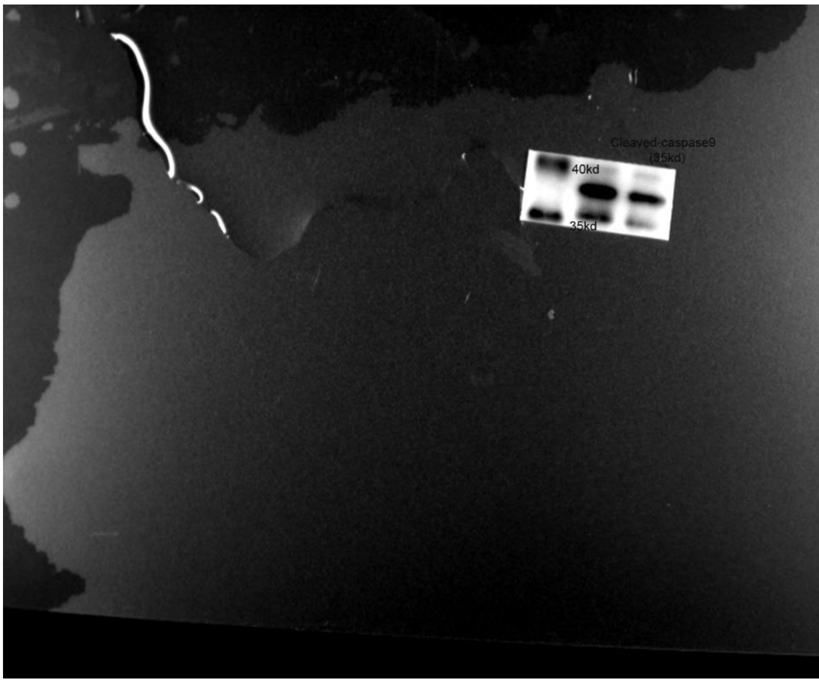

Figure 3G

786-O E-cadherin

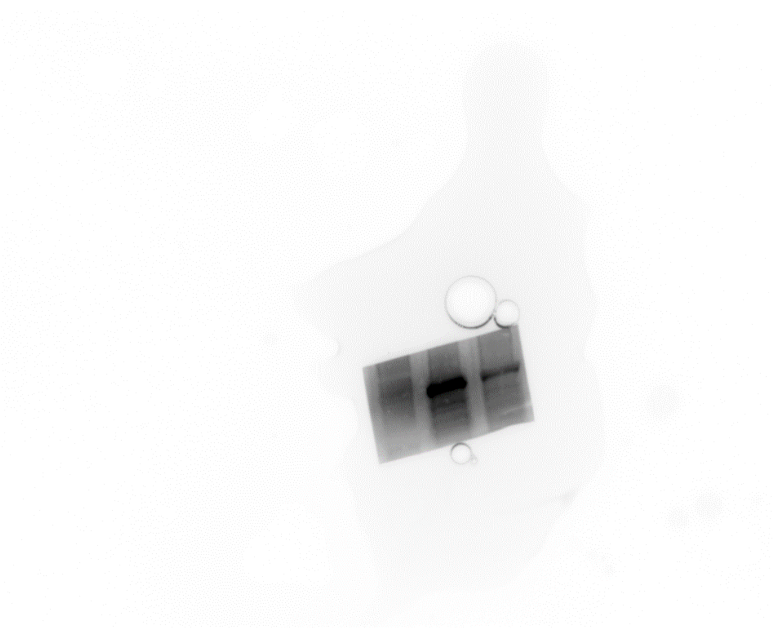

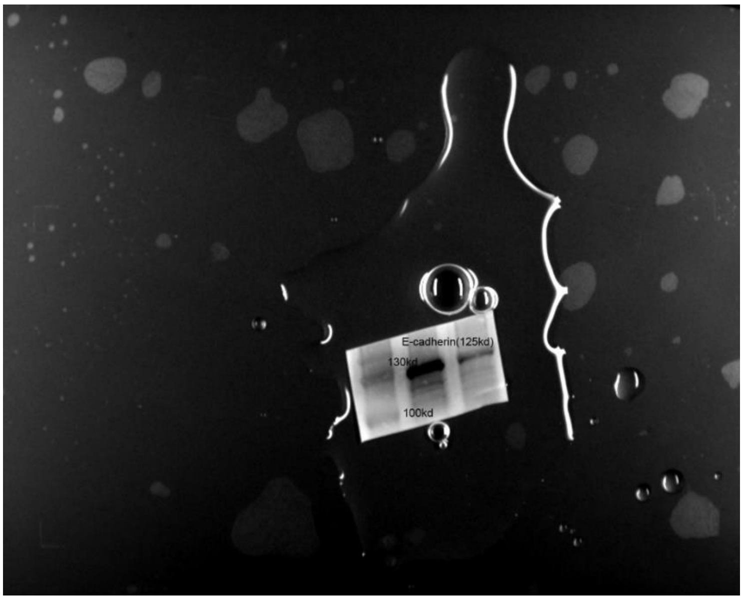

786-O GADPH

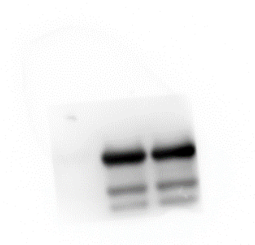

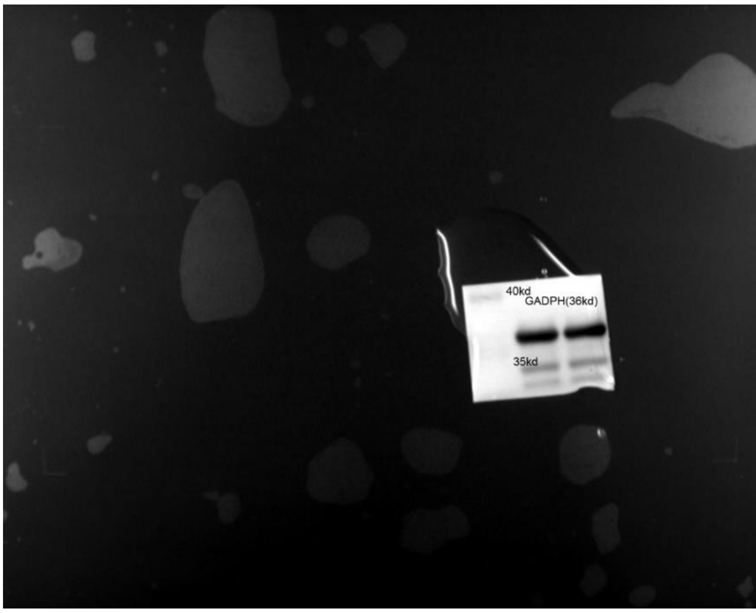

786-O N-cadherin

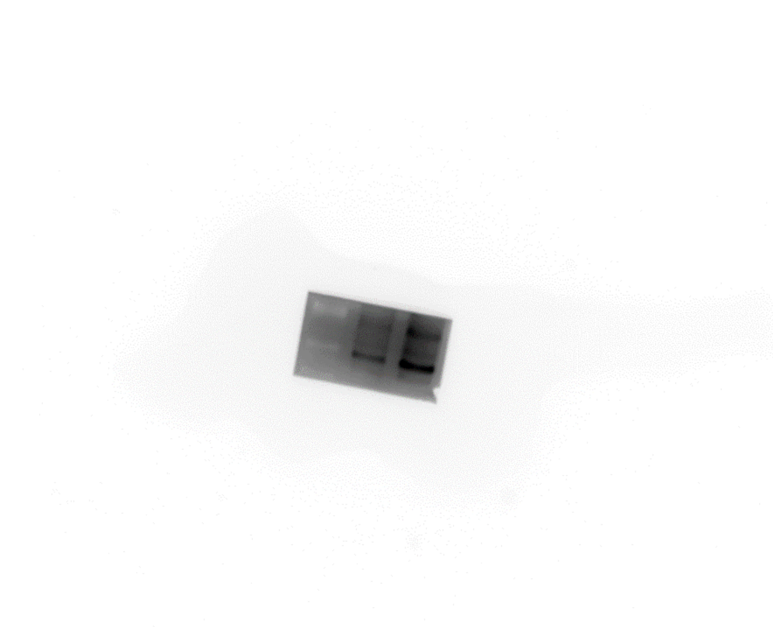

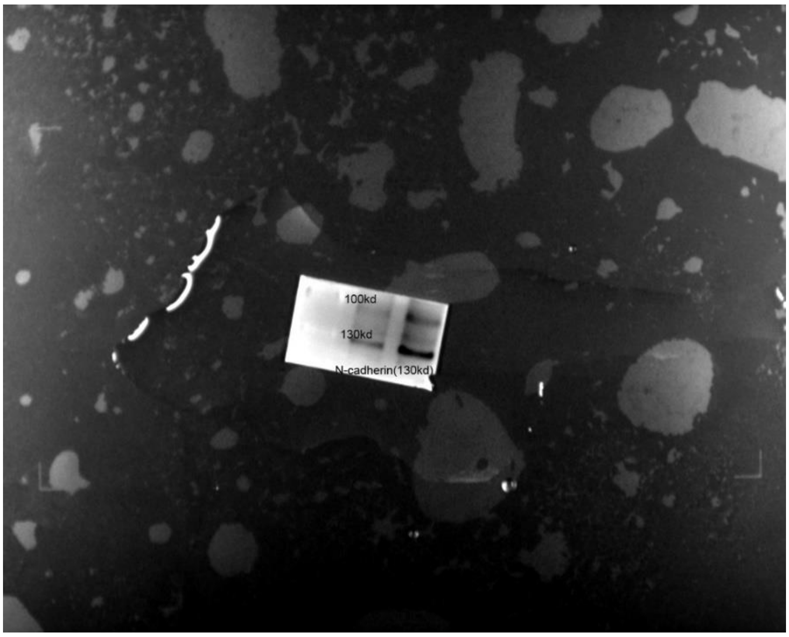

786-O Vimentin

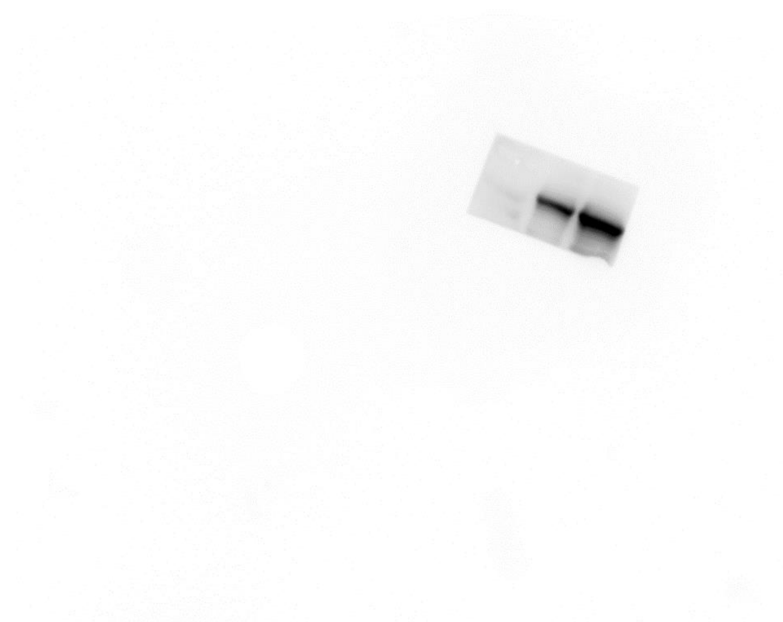

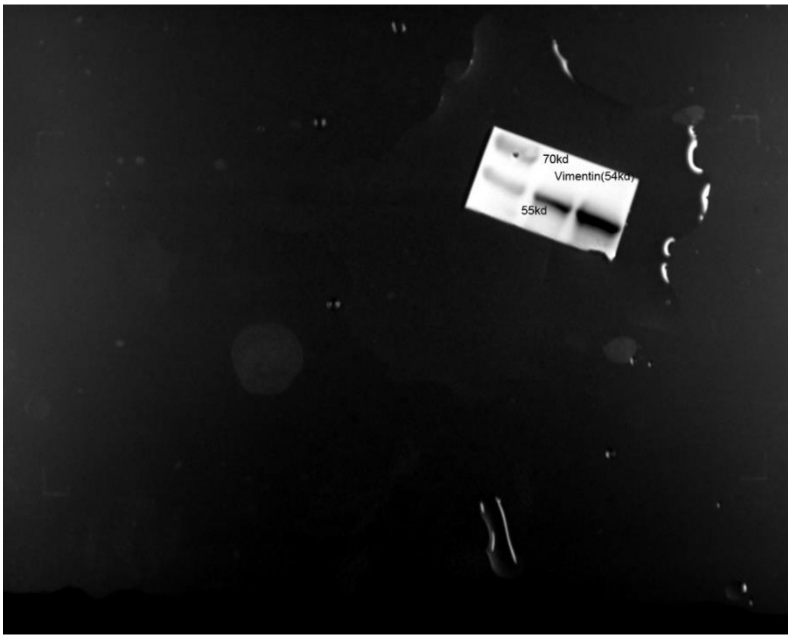

ACHN E-cadherin

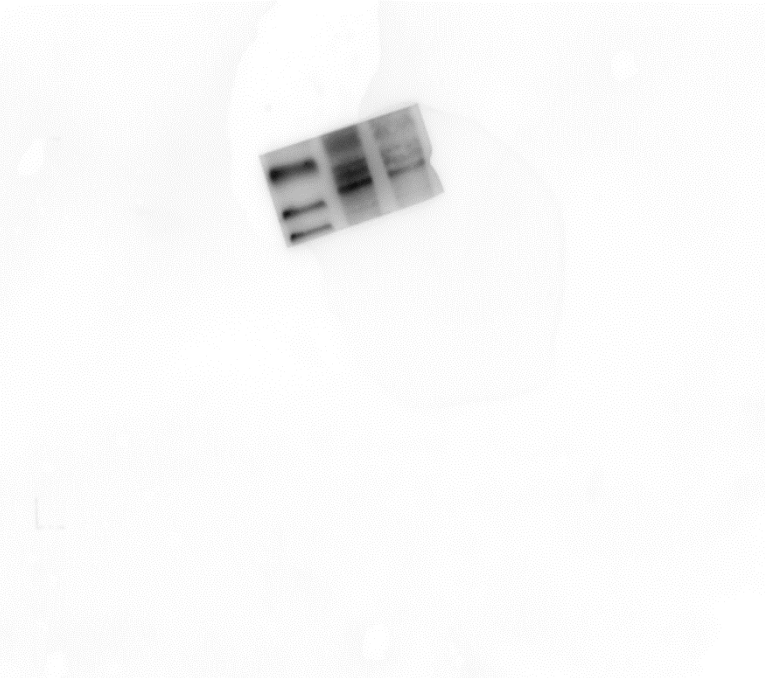

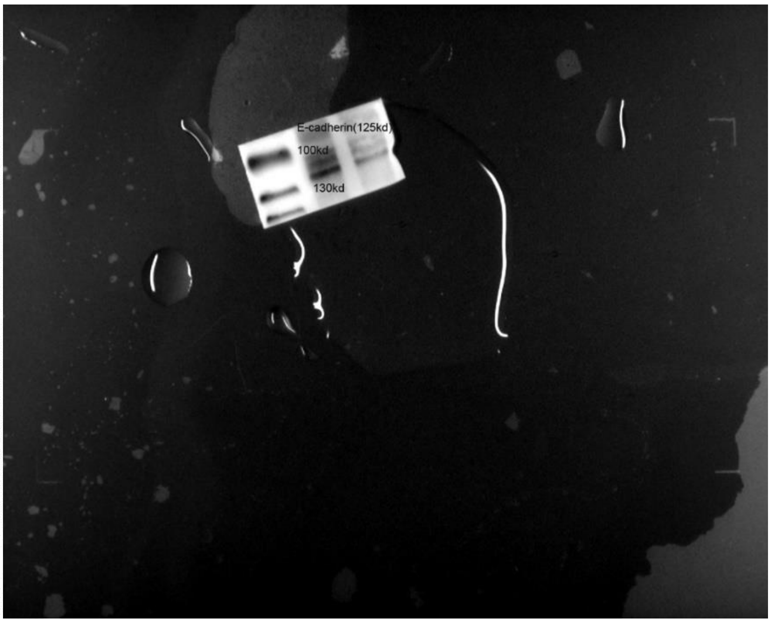

ACHN GADPH

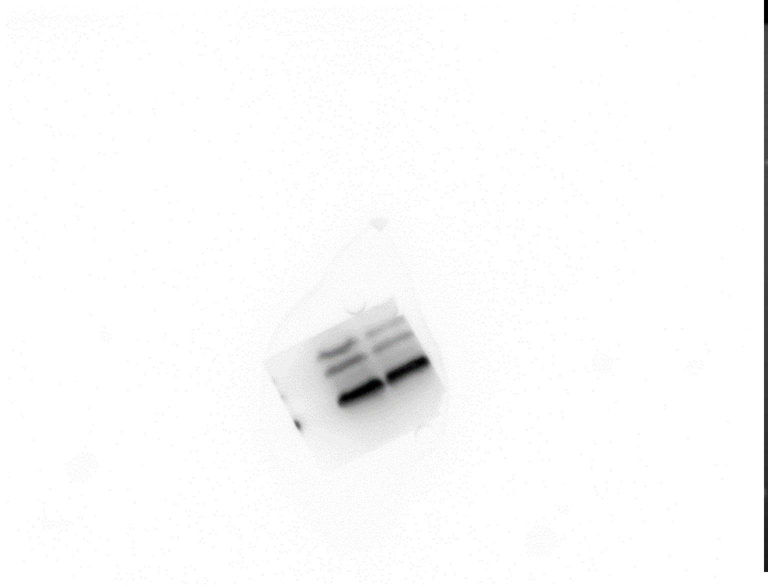

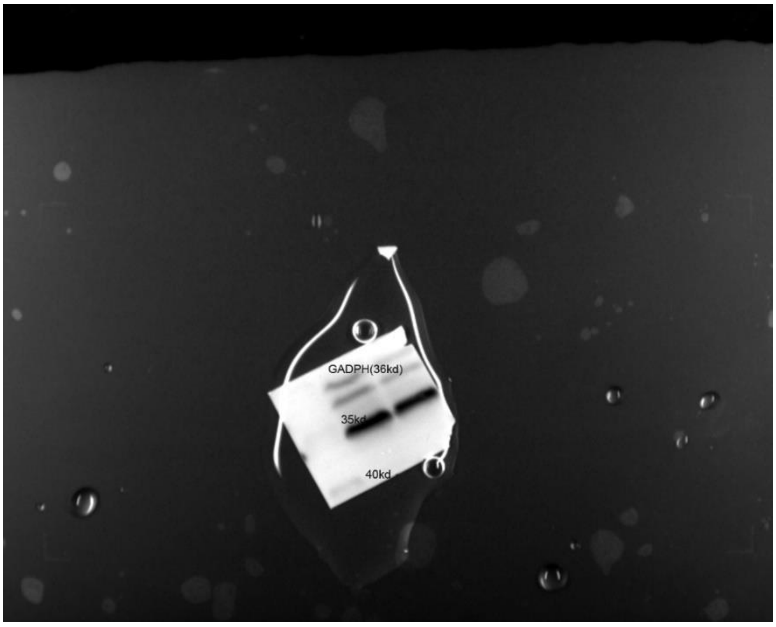

ACHN N-cadherin

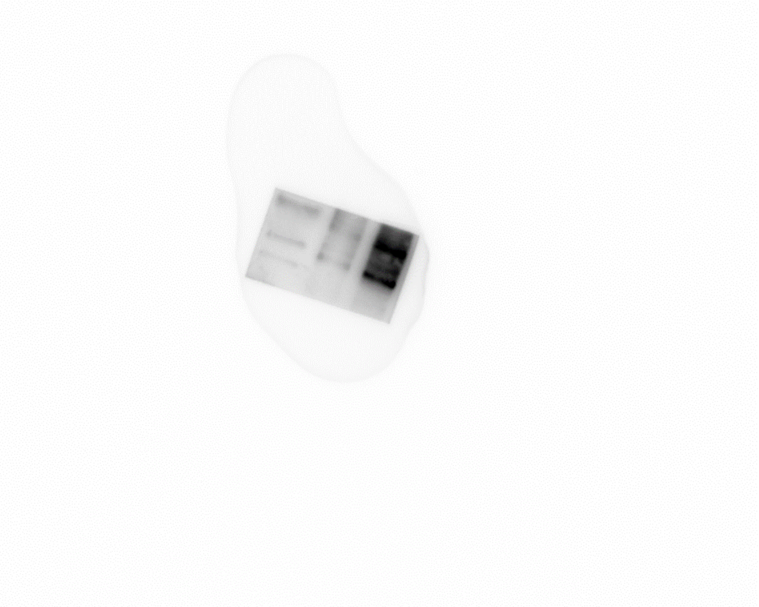

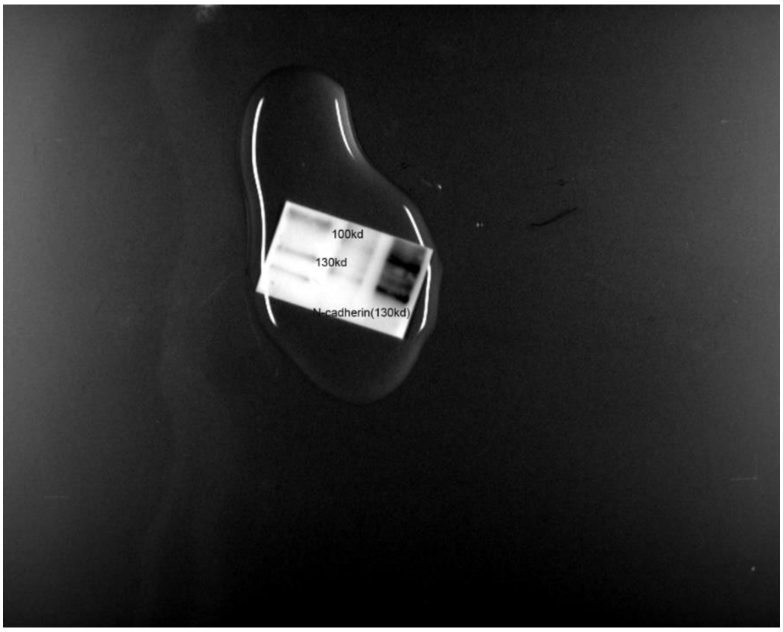

ACHN Vimentin

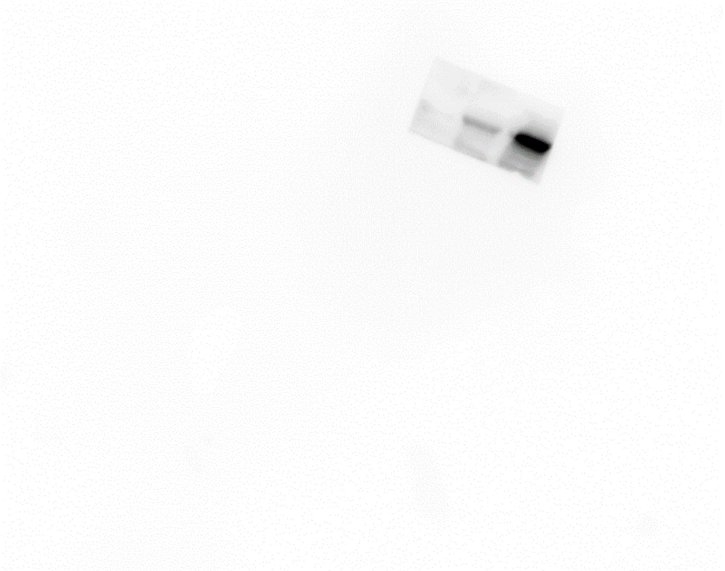

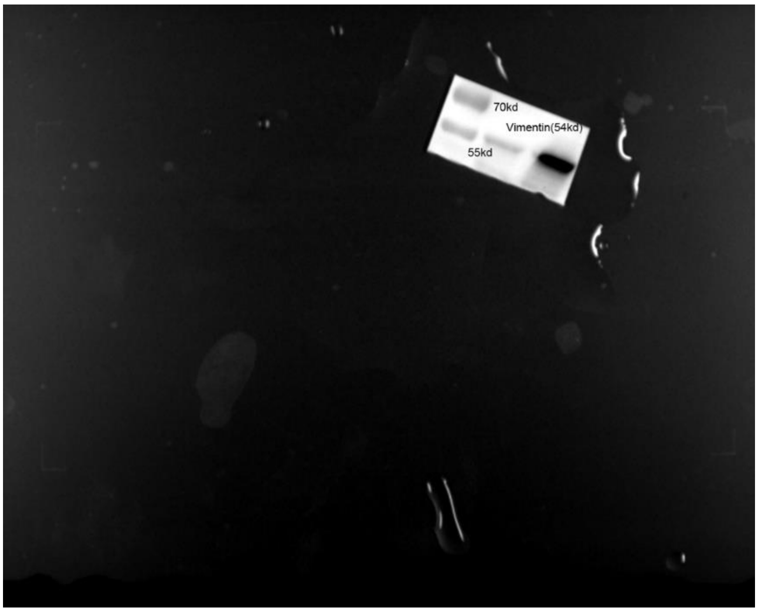

Figure 5F

786-O

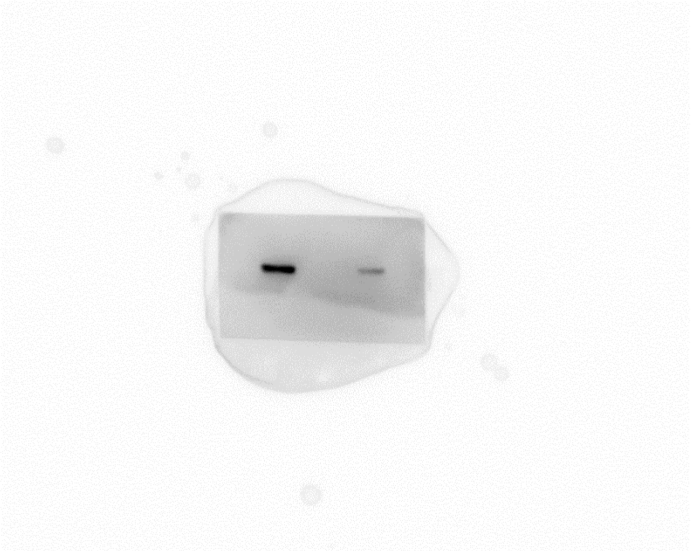

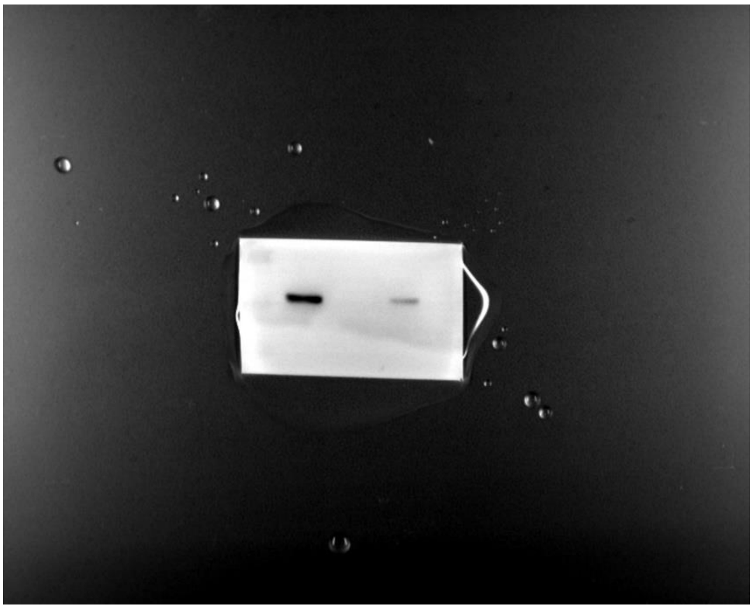

ACHN

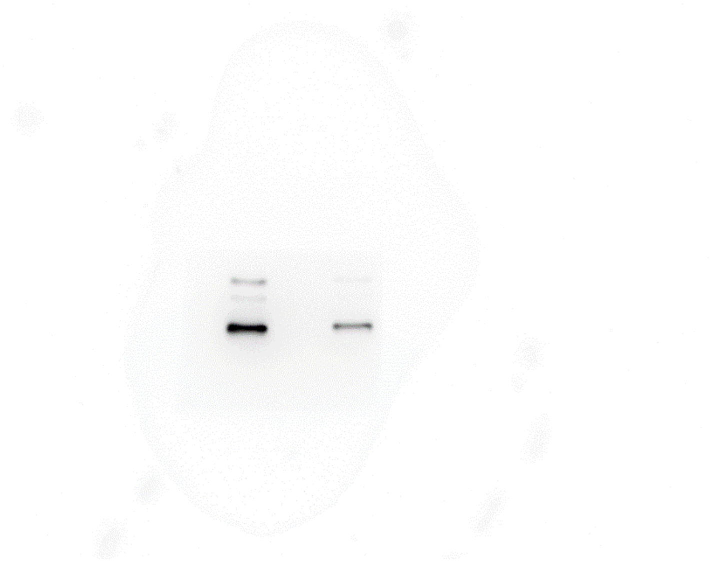

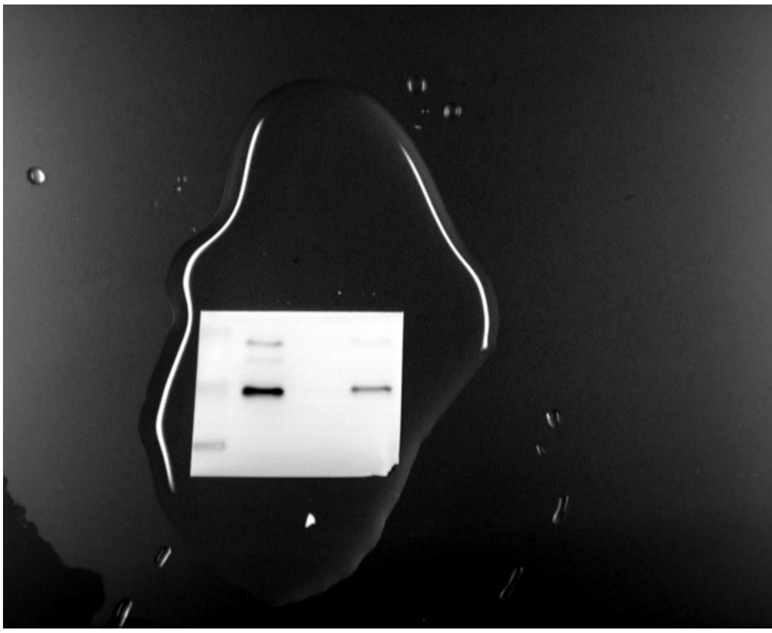

Figure 6G

786-O CCND1

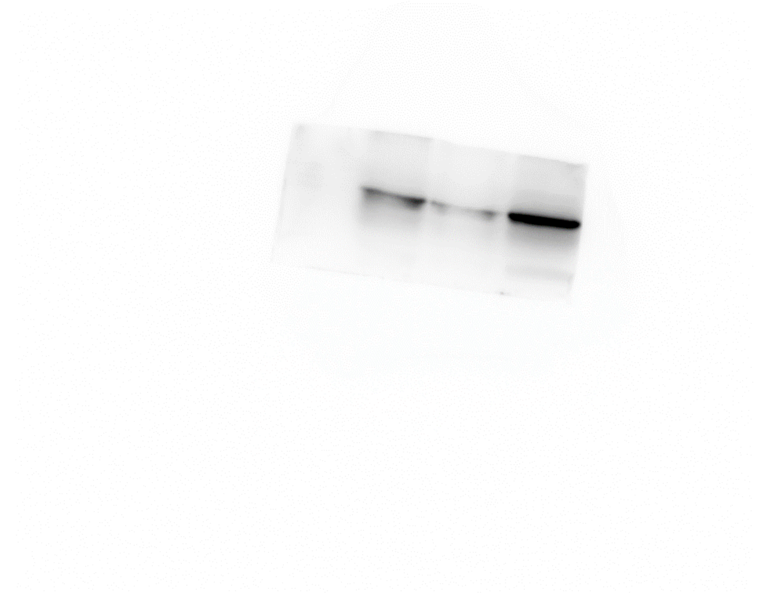

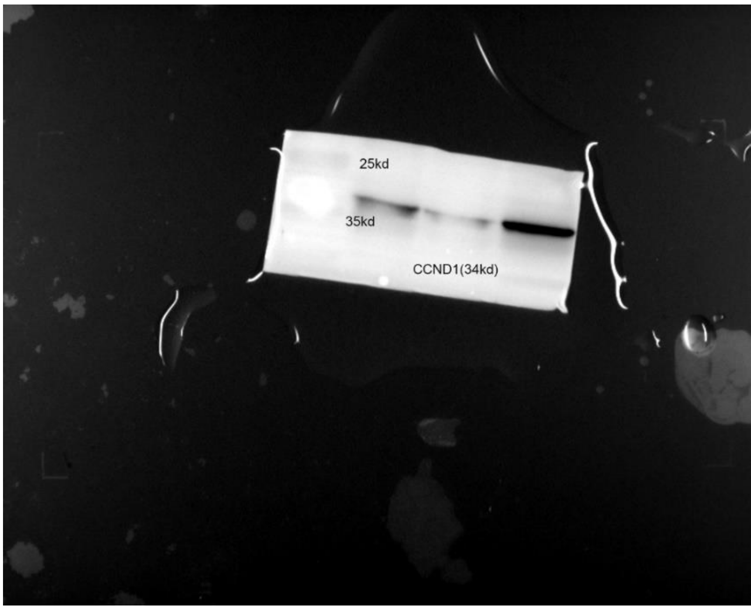

786-O TUBLIN

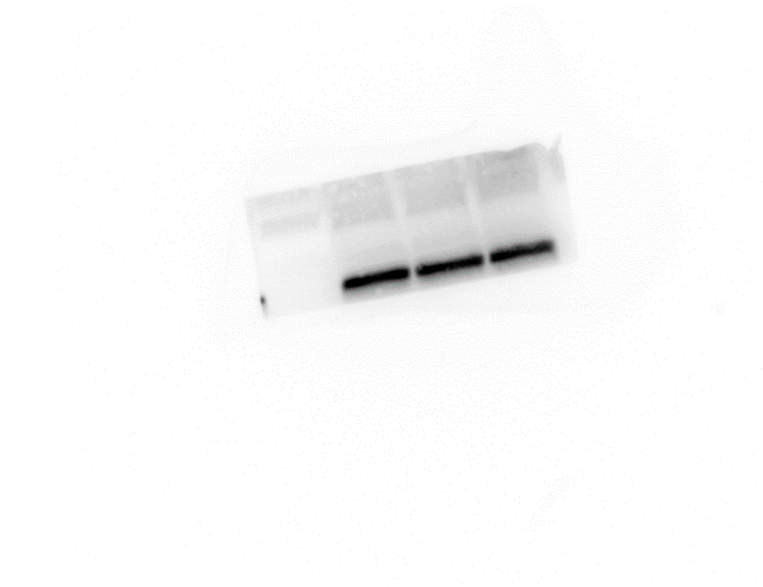

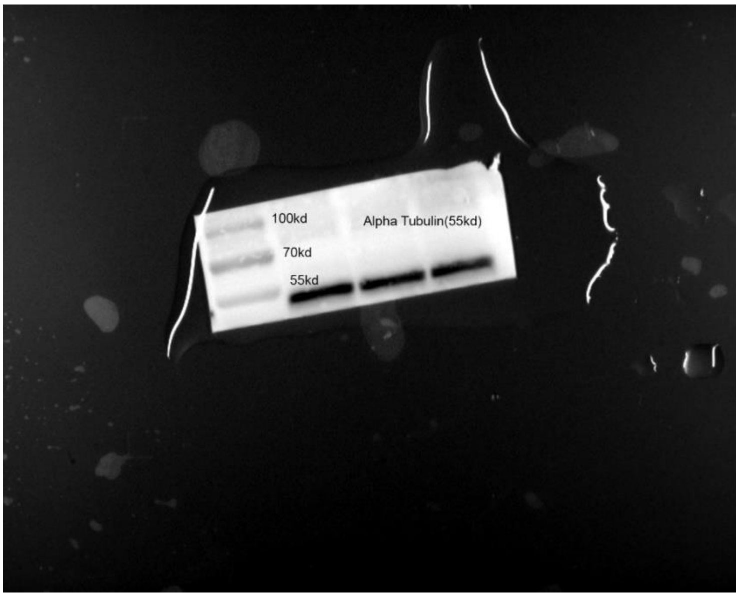

ACHN CCND1

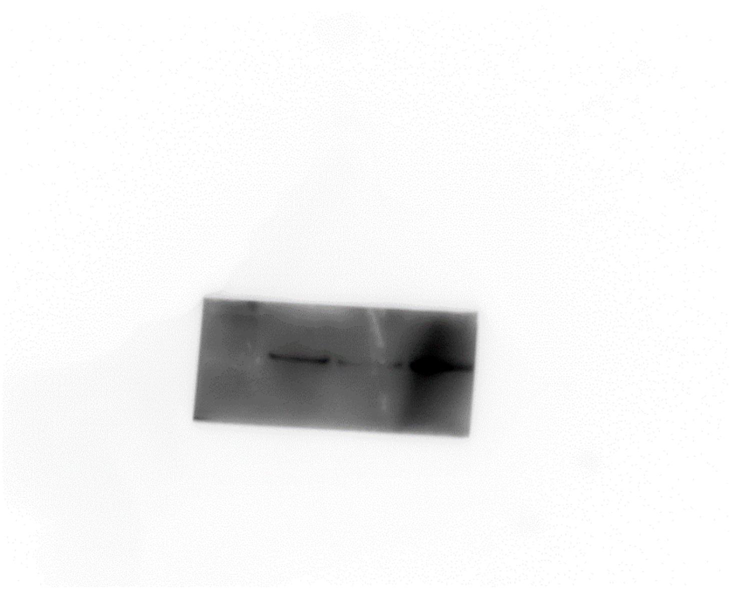

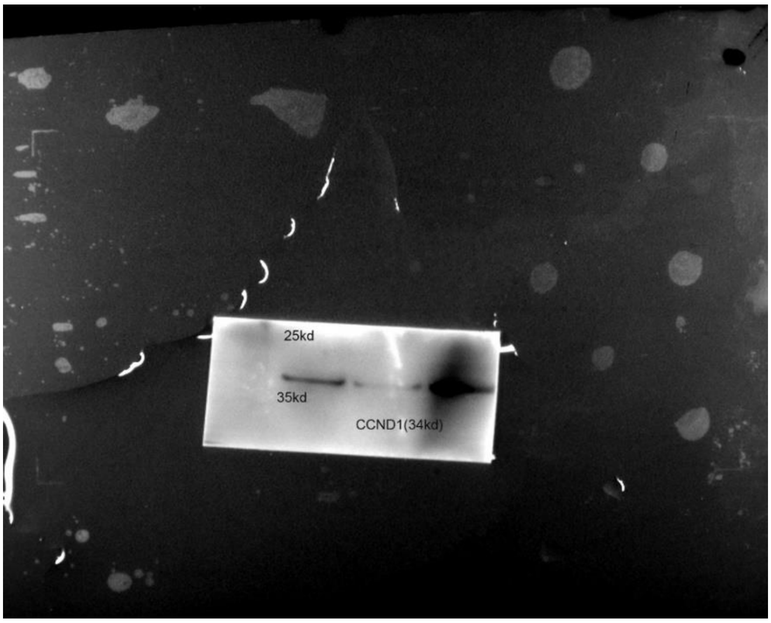

ACHN TUBLIN

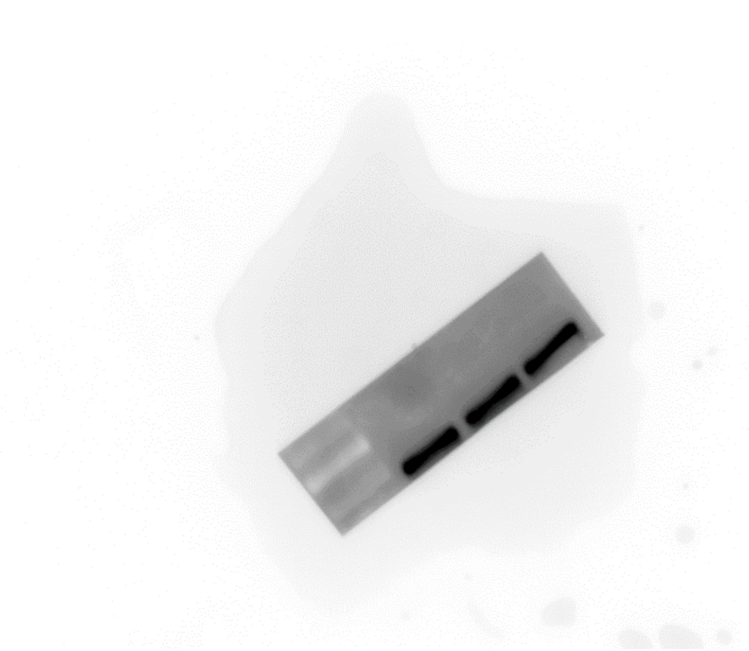

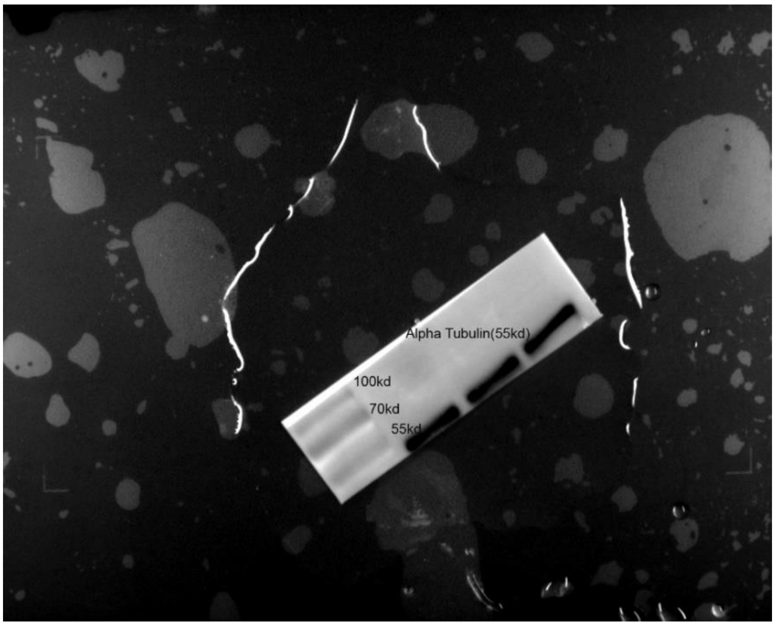

Figure 6I

786-O CCND1

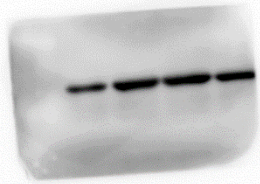

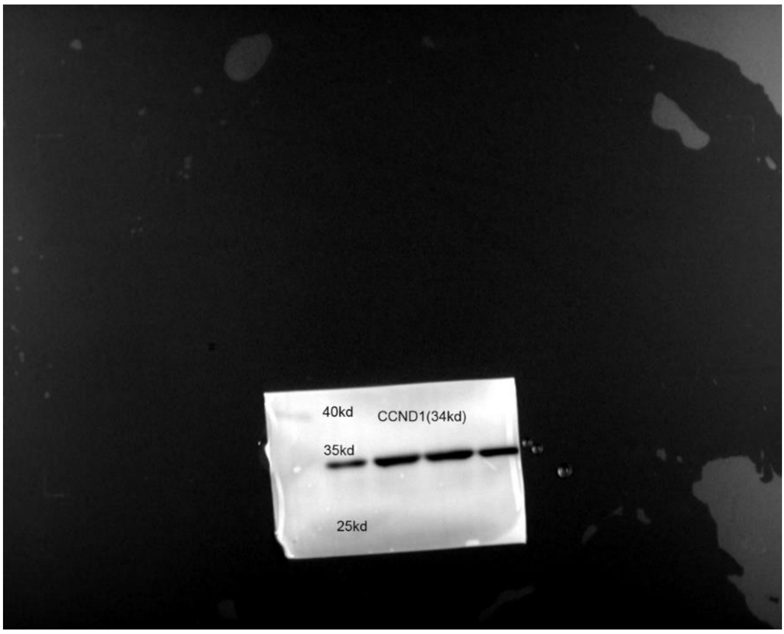

786-O TUBLIN

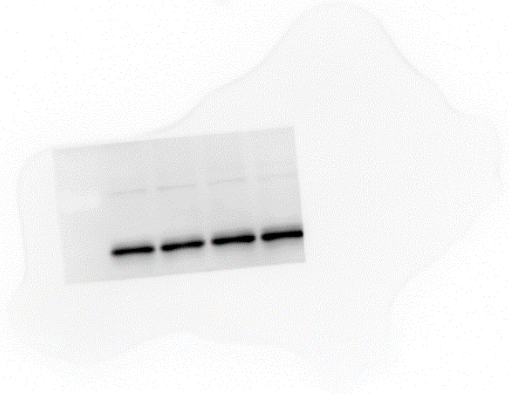

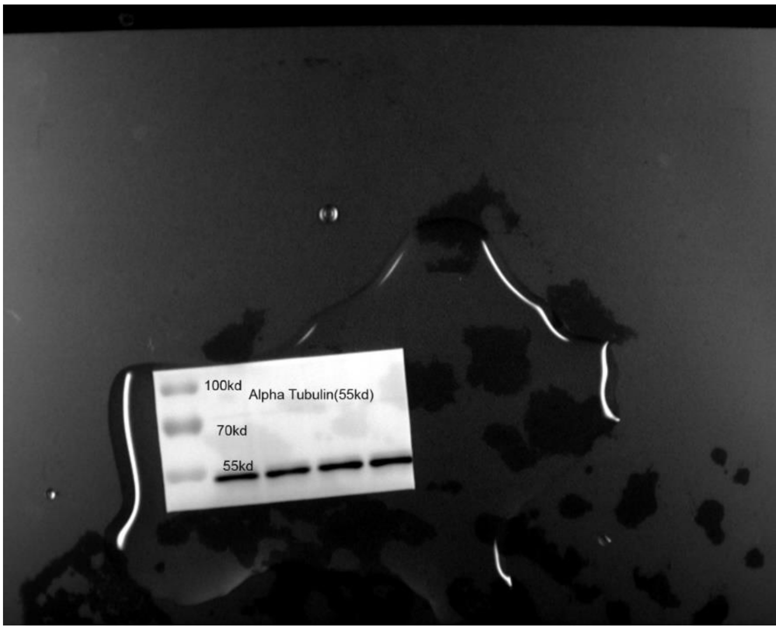

ACHN CCND1

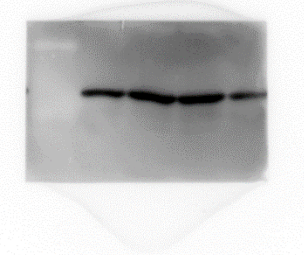

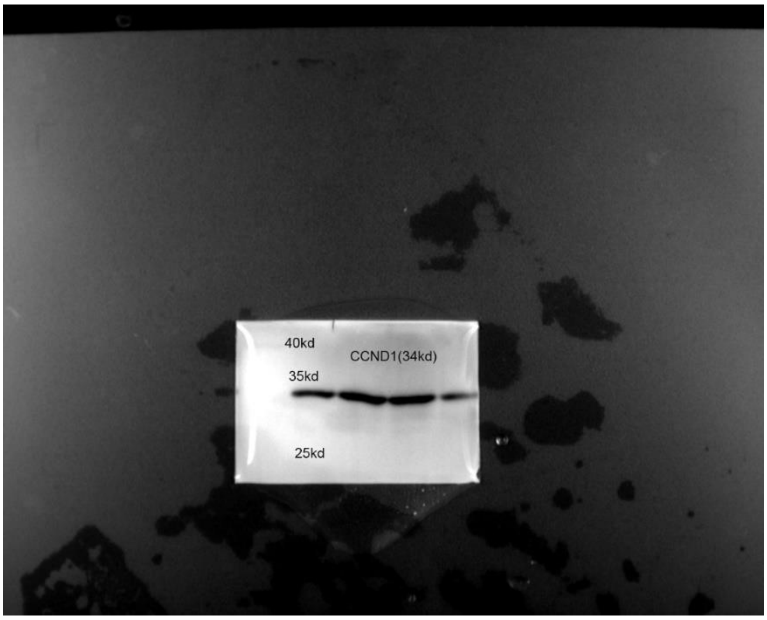

ACHN TUBLIN

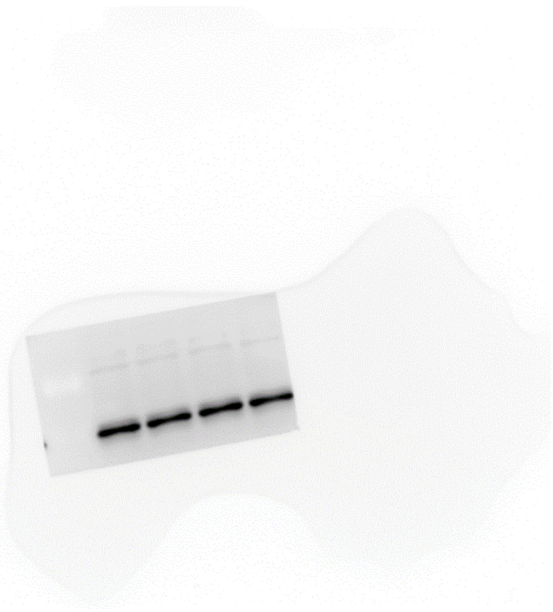

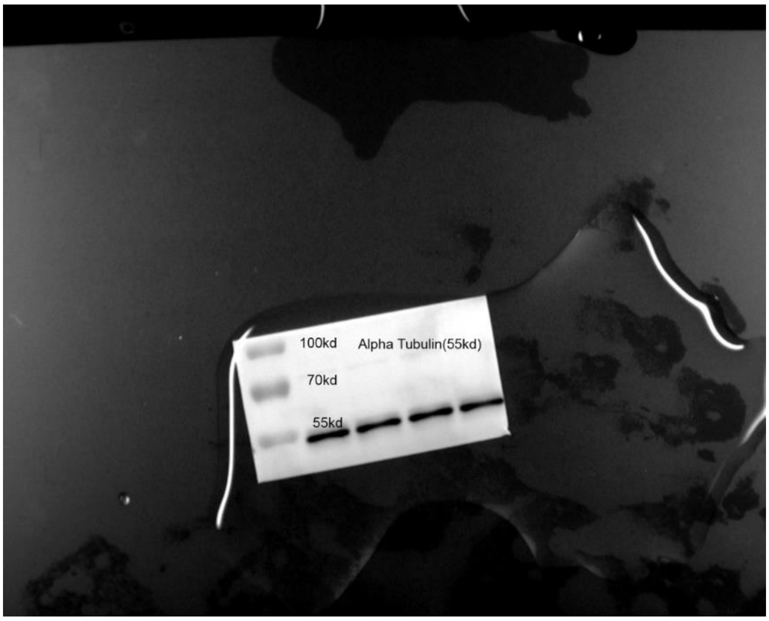

Supplement: Supplementary file 1 [file cancers-15-00123-s001.zip › cancers-1977369-file-S1.pdf]
