# Supplementary material for: Tumor Cell-Derived Exosomal circ-PRKCI Promotes Proliferation of Renal Cell Carcinoma via Regulating miR-545-3p/CCND1 Axis
Source: Cancers (Basel). 2022 Dec 25;15(1):123. doi: 10.3390/cancers15010123 (PMC9817713; doi:10.3390/cancers15010123)
Supplement: Supplementary file 1 [file cancers-15-00123-s001.zip › cancers-1977369-Tables and Figures.pdf]

Supplementary Table S1. The primer sequences of genes

Gene name	Forward (5′ to 3′)	Reverse (5′ to 3′)
circ-PRKCI	ACGATGACATTGTG AGGAAGATTG	ATGATATCCCCGCGGTAGTAG
miR-545-3p	TCGGCAGGTCAGCA AACATTTA	GTCGTATCCAGTGCAGGGTCCGAGGTATTC GCACTGGATACGACGCACAC
CCND1	ATCAAGTGTGACCC GGACTG	ATCAAGTGTGACCCGGACTG
GADPH	CCAGGTGGTCTCCT CTGA	GCTGTAGCCAAATCGTTGT
U6	CTCGCTTCGGCAGC ACA	AACGCTTCACGAATTTGCGT

Supplementary Table S2. Information of antibodies

Antibody name	Dilution	Brand	Catalog number
GM130	1:1000	CST, Shanghai, China	#74220
CD54	1:1000	CST, Shanghai, China	#74220
Annexin	1:1000	CST, Shanghai, China	#74220
CD63	1:1000	CST, Shanghai, China	#74220
BCL-2	1:5000	Proteintech, Shanghai, China	12789-1-AP
Cleaved-caspase3	1:500	Proteintech, Shanghai, China	19677-1-AP
Cleaved-caspase9	1:500	Proteintech, Shanghai, China	10380-1-AP
Beta Actin	1:10000	Proteintech, Shanghai, China	20536-1-AP
E-cadherin	1:5000	Proteintech, Shanghai, China	20874-1-AP
N-cadherin	1:2000	Proteintech, Shanghai, China	22018-1-AP
Vimentin	1:5000	Proteintech, Shanghai, China	10366-1-AP
GADPH	1:20000	Proteintech, Shanghai, China	10494-1-AP
CCND1	1:5000	Proteintech, Shanghai, China	60186-1-Ig
Alpha Tubulin	1:10000	Proteintech, Shanghai, China	11224-1-AP

Supplementary Table S3. Thirteen intersective genes between four databases

Gene
ARIH1
ZCCHC3
RBPJ
PLAG1
PEX5L
AGO4
FAM13A
TUBB
SLC16A9
DISC1
CCND1
SCARF1
TPM3

Supplementary Figure S1

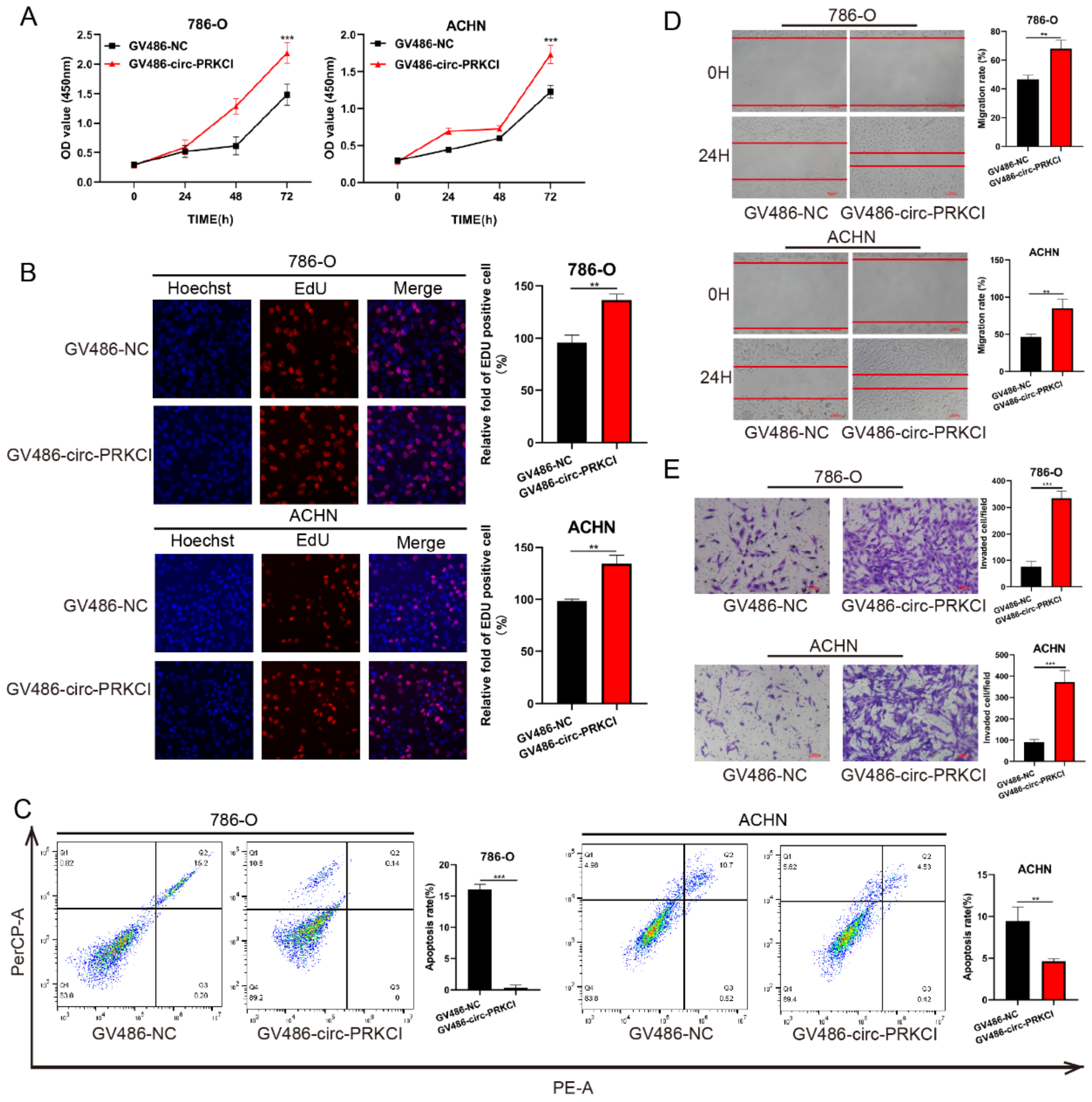

Figure S1. Overexpression of circ-PRKCI enhanced RCC proliferation, migration, invasion, and apoptosis inhibition. (A and B) CCK-8 and EdU assays showed the cell proliferation ability was enhanced after upregulating circ-PRKCI. (C) Flow cytometry analysis indicated cell apoptosis was inhibited after transfected with GV486-circ-PRKCI. (D and E) Cell scratch and Transwell assays showed the increased migration and invasion ability of transfected RCC cells. (* $p < 0.05$, ** $p < 0.01$, *** $p < 0.001$)

Supplementary Figure S2

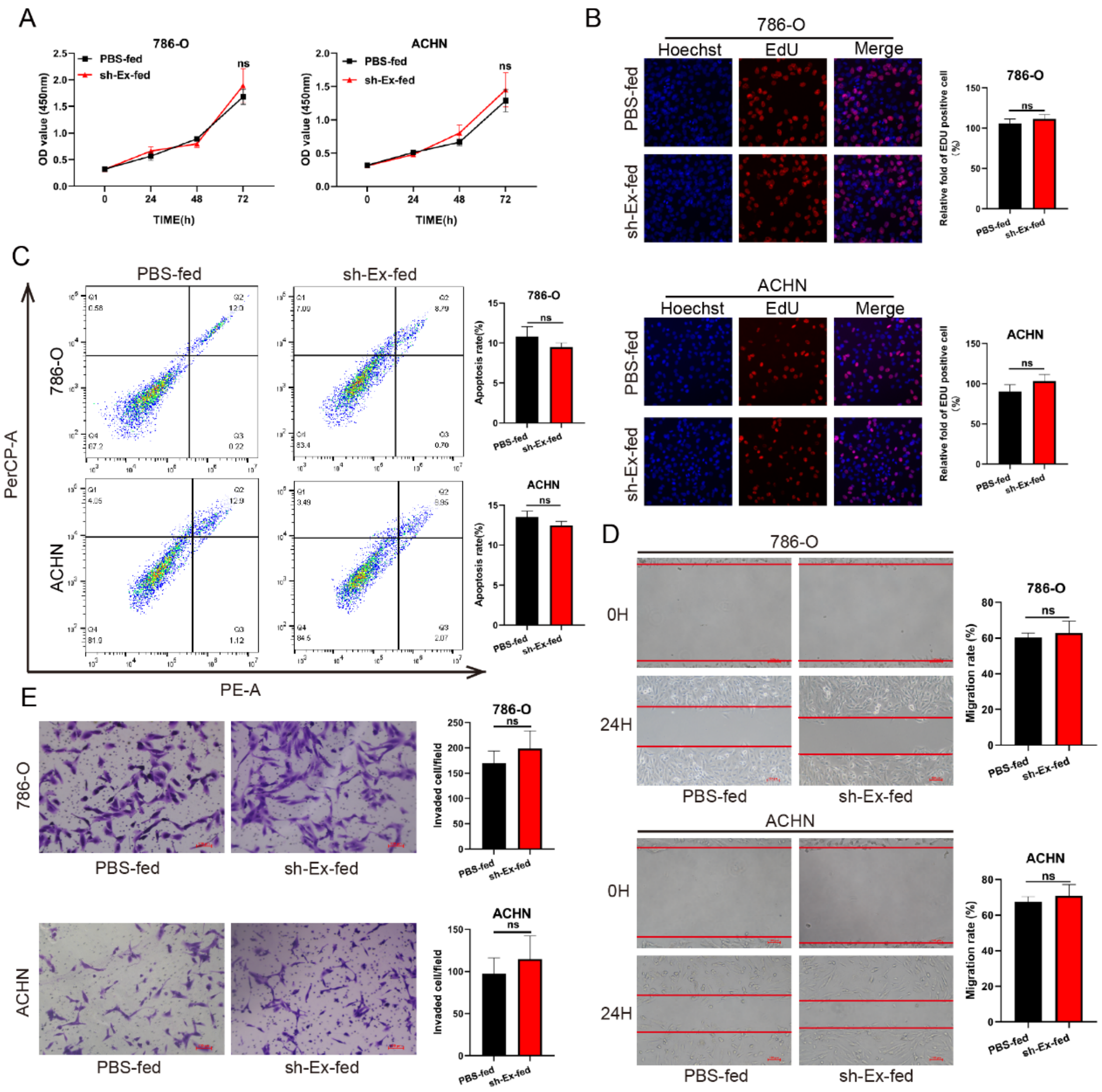

Figure S2. Little effect of sh-Ex on RCC proliferation, apoptosis, migration and invasion. (A and B) Cell proliferation of 786-O and ACHN treated with PBS or sh-Ex was detected by CCK8 and EdU assay. (C) Cell apoptosis was detected by flow cytometry analysis. (D) Cell scratch assay for migration ability of 786-O and ACHN treated with PBS or sh-Ex. (E) Transwell assay for invasion ability of 786-O and ACHN treated with PBS or sh-Ex. (ns: no significance)
